# Loss of MLKL ameliorates liver fibrosis by inhibiting hepatocyte necroptosis and hepatic stellate cell activation

**DOI:** 10.7150/thno.71400

**Published:** 2022-07-04

**Authors:** Ren Guo, Xiaohui Jia, Zhenbin Ding, Gang Wang, Mengmeng Jiang, Bing Li, Shanshan Chen, Bingqing Xia, Qing Zhang, Jian Liu, Ruting Zheng, Zhaobing Gao, Xin Xie

**Affiliations:** 1 State Key Laboratory of Drug Research, National Center for Drug Screening, Shanghai Institute of Materia Medica, Chinese Academy of Sciences, Shanghai 201203, China; 2 University of Chinese Academy of Sciences, No.19A Yuquan Road, Beijing 100049, China; 3 Department of Liver Surgery and Transplantation, Liver Cancer Institute, Zhongshan Hospital, Fudan University, Shanghai 200031, China; 4 Key Laboratory of Carcinogenesis and Cancer Invasion of Ministry of Education, Shanghai 200031, China; 5 Shanghai Xuhui Central Hospital, Zhongshan-Xuhui Hospital, Fudan University, Shanghai 200031, China; 6 Department of Pharmaceutics, School of Pharmacy, Fudan University, Shanghai 201203, China; 7 School of Life Science and Technology, ShanghaiTech University, Shanghai 201210, China; 8 School of Pharmaceutical Science and Technology, Hangzhou Institute for Advanced Study, University of Chinese Academy of Sciences, Hangzhou 310024, China; 9 CAS Key Laboratory of Receptor Research, Shanghai Institute of Materia Medica, Chinese Academy of Sciences, Shanghai 201203, China

**Keywords:** Mixed lineage kinase domain-like protein, MLKL, liver fibrosis, liver parenchymal cells, hepatocyte, hepatic stellate cells, necroptosis

## Abstract

**Background:** Liver fibrosis affects millions of people worldwide without an effective treatment. Although multiple cell types in the liver contribute to the fibrogenic process, hepatocyte death is considered to be the trigger. Multiple forms of cell death, including necrosis, apoptosis, and necroptosis, have been reported to co-exist in liver diseases. Mixed lineage kinase domain-like protein (*MLKL*) is the terminal effector in necroptosis pathway. Although necroptosis has been reported to play an important role in a number of liver diseases, the function of MLKL in liver fibrosis has yet to be unraveled.

**Methods and Results:** Here we report that MLKL level is positively correlated with a number of fibrotic markers in liver samples from both patients with liver fibrosis and animal models. Mlkl deletion in mice significantly reduces clinical symptoms of CCl_4_- and bile duct ligation (BDL) -induced liver injury and fibrosis. Further studies indicate that *Mlkl*^-/-^ blocks liver fibrosis by reducing hepatocyte necroptosis and hepatic stellate cell (HSC) activation. AAV8-mediated specific knockdown of *Mlkl* in hepatocytes remarkably alleviates CCl_4_-induced liver fibrosis in both preventative and therapeutic ways.

**Conclusion:** Our results show that MLKL-mediated signaling plays an important role in liver damage and fibrosis, and targeting MLKL might be an effective way to treat liver fibrosis.

## Introduction

Hepatic fibrosis, a reversible wound healing response, occurs in diverse etiologies, including hepatotoxicity, viral hepatitis, autoimmune disorders, metabolic disorders, nonalcoholic steatohepatitis (NASH), and cholestatic liver diseases 1, 2. Repeated liver injury and aberrant tissue healing may result in liver fibrosis. If liver injury ceases, the reversal of early liver fibrosis can occur 3, 4. Advanced liver fibrosis usually progresses into irreversible liver cirrhosis, portal hypertension, and hepatic failure, which will eventually conduce to liver cancer 5. To date, there is no approved pharmacological therapy for liver fibrosis worldwide, highlighting an urgent medical demand to investigate novel and effective therapeutics to surmount liver fibrosis 6.

Human liver fibrosis may have various aetiologies, but common key cellular and molecular events have been summarized 7, 8. Liver 'insult' from different origins, including viral, toxic, metabolic, or autoimmune, damage hepatocytes, leads to the release of inflammatory mediators to activate Kupffer cells, the resident liver macrophages, which in turn secrete inflammatory cytokines and chemokines that attract immune cells 9. Neutrophils are often recruited to the damaged liver and act as the first responder to clear damaged hepatocytes 10. Neutrophils release extensive chemoattractants, which elicit a strong proinflammatory effect 11. These further contribute to hepatocyte injury and also promote the recruitment of a large number of circulating monocyte-derived macrophages, which have dual functions in liver fibrosis progression and its resolution 12. During the progression of fibrosis, inflammatory factors facilitate the recruitment of monocyte-derived LY6C^high^ macrophages to the liver 12, 13, where they produce large amount of cytokines and chemokines. The quiescent hepatic stellate cells (HSCs) become activated and differentiate into myofibroblast-like cells, which secrete excessive extracellular matrix proteins, and lead to the formation of fibrous scar 14.

Although multiple cell types in the liver contribute to the fibrogenic process 15, hepatocyte death is considered to be the trigger 16, 17. Multiple forms of cell death, including necrosis, apoptosis, and necroptosis, have been reported to co-exist in liver diseases 15, 18. In contrast to necrosis, a largely unregulated consequence of physicochemical stress and a highly immunogenic death, and apoptosis, a highly controlled biochemical process and a low inflammatory death, necroptosis incorporates features of necrosis and apoptosis. Necroptosis uses the same upstream molecular machinery as apoptosis but leads to cellular leakage as a result of organelle and cellular swelling 19, 20. Key regulators of necroptosis include receptor interacting protein kinase 1 and 3 (RIP1 and RIP3), and downstream effector MLKL (Mixed lineage kinase domain-like protein), which upon phosphorylation, forms oligomers or polymers and binds to negatively charged phospholipids, causing cell membrane destruction and subsequent cell death 21, 22.

Accumulating evidences indicate that necroptosis is a trigger of inflammation, and plays a critical role in the pathogenesis of liver diseases 23. Previous studies have shown that RIP1 kinase activity promotes steatohepatitis by mediating macrophage cell death and inflammation 24. Ablation of *Rip3* prevents ethanol-induced liver injury and CCl_4_-induced liver fibrosis 25, 26, however, additional results have suggested that RIP3 protects mice from high-fat-diet (HFD) induced liver injury 27. Deficiency of *Mlkl* alleviates hepatic insulin resistance and glucose intolerance 28. Knockout of *Mlkl* has a protective effect on NASH induced by high fat, fructose, and cholesterol diet (FFC) through inhibition of hepatocyte autophagy 29.

Here we sought to elucidate the function of MLKL in liver fibrosis and underlying mechanisms.

## Result

### Up-regulation of *Mlkl* in liver is associated with liver fibrosis

**Figure 1:**
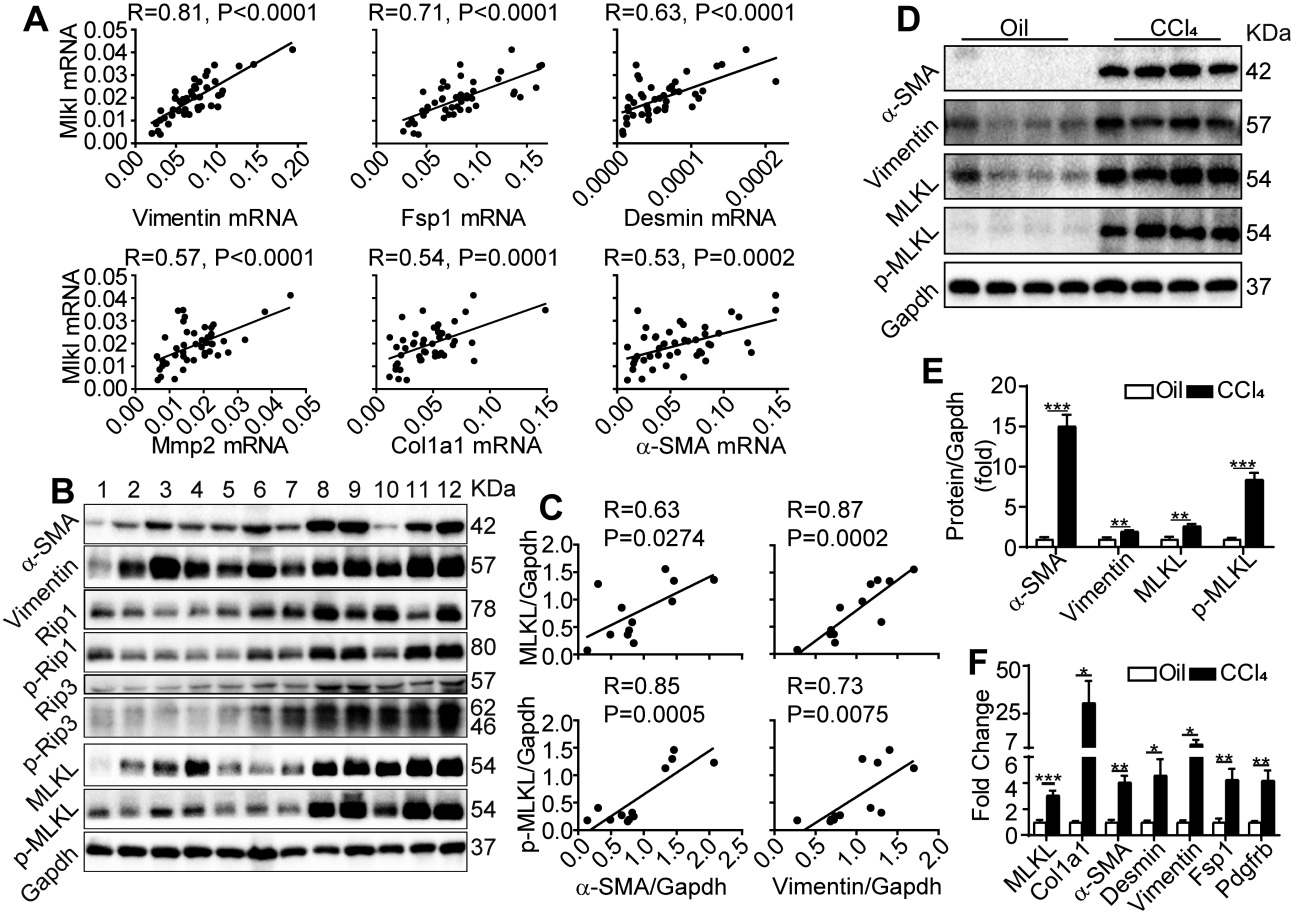
** MLKL level in the liver is positively correlated with human and mice liver fibrosis.** (A) Quantitative RT-PCR analysis of the mRNA levels of *Mlkl* and fibrosis markers *Vimentin*,* Fsp1*,* Desmin*, *Mmp2*,* Col1a1*, and *α-SMA* in the liver biopsy samples of patients with fibrosis/cirrhosis (n=45). Pearson's correlation coefficient and statistical significance are listed in the plots. (B) Western blot analysis of RIP1, RIP3, MLKL, and their phosphorylated forms, together with fibrosis markers α-SMA and Vimentin in the liver biopsy samples of patients with fibrosis/cirrhosis (n=12, each lane represents one patient sample). Gapdh was used as loading control. (C) The Pearson's correlations among the protein levels of α-SMA, Vimentin, MLKL, and p-MLKL in (B). The protein levels were normalized to Gapdh in the same sample. (D) Western blot analysis of α-SMA, Vimentin, MLKL and p-MLKL in the liver of mice treated with CCl_4_ or vehicle (oil) three times a week for 8 weeks (n=4, each lane represents one animal sample). Gapdh was used as loading control. (E) Quantification of the blots in (D). The protein levels were normalized to Gapdh in the same sample, then normalized to vehicle control. (F) Quantitative RT-PCR analysis of *Mlkl* and fibrosis genes *Col1a1, a-SMA, Desmin, Vimentin, Fsp1, and Pdgfrb* in liver samples of mice treated with oil or CCl_4_ (n=4). All data are shown as Means ± SEM, *P < 0.05, **P< 0.01, ***P< 0.001 (Student's t-test).

We first examined the expression of fibrosis-related genes and *Mlkl* in liver biopsy samples collected from patients with cirrhosis. Quantitative RT-PCR revealed that *Mlkl* expression in the cirrhotic livers showed a very strong positive correlation with the expression of *Vimentin* (with Pearson's correlation coefficient R=0.81, P<0.0001) and a strong to moderate positive correlation (with R ranging from 0.71~0.53, P≤0.0002) with a number of fibrotic genes including *Fsp1, Desmin, Mmp2, Col1a1* and *α-SMA* (Figure 1A). Moreover, western blot analysis of patient liver samples also indicated that all components of necrosome complex including RIP1/p-RIP1, RIP3/p-RIP3 and MLKL/p-MLKL were upregulated in cirrhotic livers (Figure 1B), and very strong to strong positive correlations between the protein levels of MLKL, p-MLKL and the fibrosis markers α-SMA and Vimentin were observed (Figure 1B-C). Furthermore, in the classical CCl_4_ induced mice liver fibrosis model, both the western blot analysis (Figure 1D-E) and quantitative RT-PCR analysis (Figure 1F) confirmed that MLKL was significantly upregulated in both the protein and mRNA levels, accompanied by the significant upregulation of a number of fibrosis markers, comparing to the vehicle control group. Importantly, the active phosphorylated form of MLKL, p-MLKL, was also increased in the CCl_4_-treated group (Figure 1D-E). Taken together, these data show a clear and strong positive correlation between MLKL and liver fibrosis, indicating that MLKL may play a role in this pathogenic process.

### Deletion of *Mlkl* in mice reduces liver inflammation in CCl_4_-induced liver injury

We then generated *Mlkl* knockout (*Mlkl^-/-^*) mice with the CRISPR-Cas9 system (Figure S1A). A founder mouse with 10 bp deletion starting from the 27th base of the transcription starting site in exon1 in one allele (Figure S1B, mouse #2), which leads to frameshift and incorrect translation from 9th amino acid and premature translation stop at 17th amino acid, was used to breed *Mlkl^-/-^* mice (Figure S1C).

**Figure 2:**
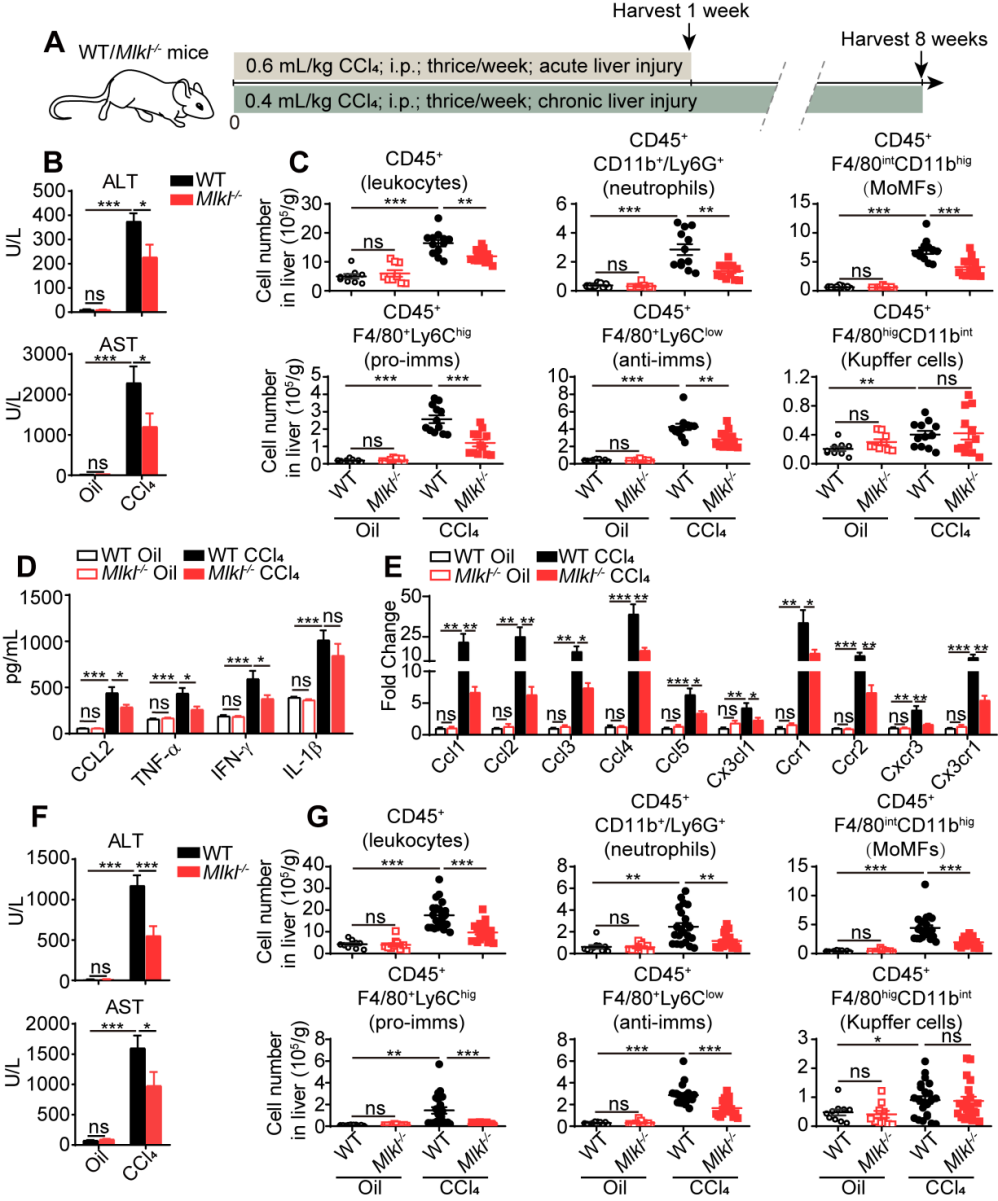
***Mlkl* deletion reduces leukocyte accumulation in the liver after CCl_4_ treatment.** (A) Schematic of the experimental design of CCl_4_-induced acute or chronic liver damage in mice. (B) The amount of ALT and AST in the serum of WT and *Mlkl*^-/-^ mice treated with oil or CCl_4_ (acute) (Oil groups, n=9; CCl_4_ groups, n=12). (C) Quantification of the numbers of total leukcocytes, neutrophils, MoMFs, pro-inflammatory macrophages (pro-imms), anti-inflammatory macrophages (anti-imms), and Kupffer cells in the liver of WT and *Mlkl*^-/-^ mice treated with oil or CCl_4_ (acute) (Oil groups, n=9; CCl_4_ groups, n=12). (D) Serum level of CCL2, TNF-α, IFN-γ, and IL-1β in WT and *Mlkl*^-/-^ mice treated with oil or CCl_4_ (acute) (Oil groups, n=9; CCl_4_ groups, n=12). (E) Quantitative RT-PCR analysis of mRNAs encoding the chemokines: *Ccl1*, *Ccl2*, *Ccl3*, *Ccl4*, *Ccl5*, *Cx3cl1,* and the related chemokine receptors: *Ccr1*, *Ccr2*, *Cxcr3*, *Cx3cr1* in the whole liver samples from WT or *Mlkl*^-/-^ mice treated with oil or CCl_4_ (acute). (Oil groups, n=9; CCl_4_ groups, n=12). (F) The amounts of ALT and AST in the serum of the WT and *Mlkl*^-/-^ mice treated with oil or CCl_4_ (chronic) (Oil groups, n=10; CCl_4_ groups: WT, n=22; KO, n=20). (G) Quantification of the numbers of total leukocytes, neutrophils, MoMFs, pro-imms, anti-imms, and Kupffer cells in the liver of WT and *Mlkl*^-/-^ mice treated with oil or CCl_4_ (chronic) (Oil groups, n=10; CCl_4_ groups: WT, n=22; KO, n=20). Data are shown as Means ± SEM, *P < 0.05, **P< 0.01, ***P< 0.001 (Student's t-test).

Previous studies have shown that inflammation is one of the critical steps linking liver injury to hepatic fibrosis. Thus we first studied immune-related events in wild type (WT) and *Mlkl*^-/-^ mice treated with CCl_4_. After acute CCl_4_ treatment (three times a week for 1 week, Figure 2A), the *Mlkl^-/-^* mice showed less hepatocellular injury, with significantly lower plasma alanine transaminase (ALT) and aspartate transaminase (AST) levels (Figure 2B). Fluorescence activated cell sorting (FACS) analysis showed that after acute CCl_4_ treatment, the numbers of total infiltrating leukocytes, neutrophils, monocyte-derived macrophages (MoMFs), pro-inflammatory macrophages (pro-imms), and anti-inflammatory macrophages (anti-imms) (Figure 2C) and the percentage of neutrophils, MoMFs and pro-imms (Figure S2A-B) were significantly lower in *Mlkl^-/-^* mice versus WT mice. Notably, the number and percentage of Kupffer cells and the percentage of anti-imms showed no significant differences in WT and *Mlkl^-/-^* mice after acute CCl_4_ treatment (Figure 2C, Figure S2A-B). Furthermore, in CCl_4_ induced acute liver damage, *Mlkl* deletion significantly reduced the elevation of chemokine CCL2 and pro-inflammatory cytokines TNF-α and IFN-γ in serum (Figure 2D). Others have reported that MLKL may contribute to the activation of NLRP3 (nucleotide-binding oligomerization domain (NOD)-like receptor protein 3) inflammasome to trigger caspase-1 processing of the pro-inflammatory cytokine IL-1β 30, but we only detected a slight but not significant reduction of IL-1β in the serum of *Mlkl^-/-^* mice after acute CCl_4_ treatment (Figure 2D). *Mlkl* deletion also remarkably decreased the mRNA level of chemokines including *Ccl1*, *Ccl2*, *Ccl3*, *Ccl4*, *Ccl5*, *Cx3cl1* and the related chemokine receptors: *Ccr1*, *Ccr2*, *Cxcr3*, *Cx3cr1* in whole liver tissues after acute CCl_4_ treatment (Figure 2E).

Previous studies have shown that CD11b^+^ cells in livers were the major pro-inflammatory cells in promoting liver fibrosis 31. So CD11b^+^ cells were purified from the livers of oil or CCl_4_ treated WT or *Mlkl^-/-^* mice and cultured *in vitro* for 24 h and the cytokines in the culture media were assessed. The results showed in WT mice, the CD11b^+^ cells collected from CCl_4_ group secreted more CCL2, TNF-α, IFN-γ, and IL-1β versus the oil group, but all cytokines except IL-1β were dramatically reduced in CD11b^+^ cells isolated from the livers of CCl_4_ treated *Mlkl^-/-^* mice (Figure S2C).

In chronic CCl_4_-induced liver damage (three times a week for 8 weeks, Figure 2A), *Mlkl^-/-^* mice also showed reduced ALT and AST levels in serum (Figure 2F), indicating less liver damage. The numbers of total infiltrating leukocytes, neutrophils, MoMFs, pro-imms, and anti-imms were all significantly lower in CCl_4_-treated *Mlkl^-/-^* mice versus CCl_4_-treated WT group (Figure 2G). Taken together, these observations suggest that knockout of *Mlkl* in mice reduces liver inflammation in CCl_4_-induced liver injury.

### Knockout of *Mlkl* in mice attenuates CCl_4_-induced hepatic fibrosis

**Figure 3:**
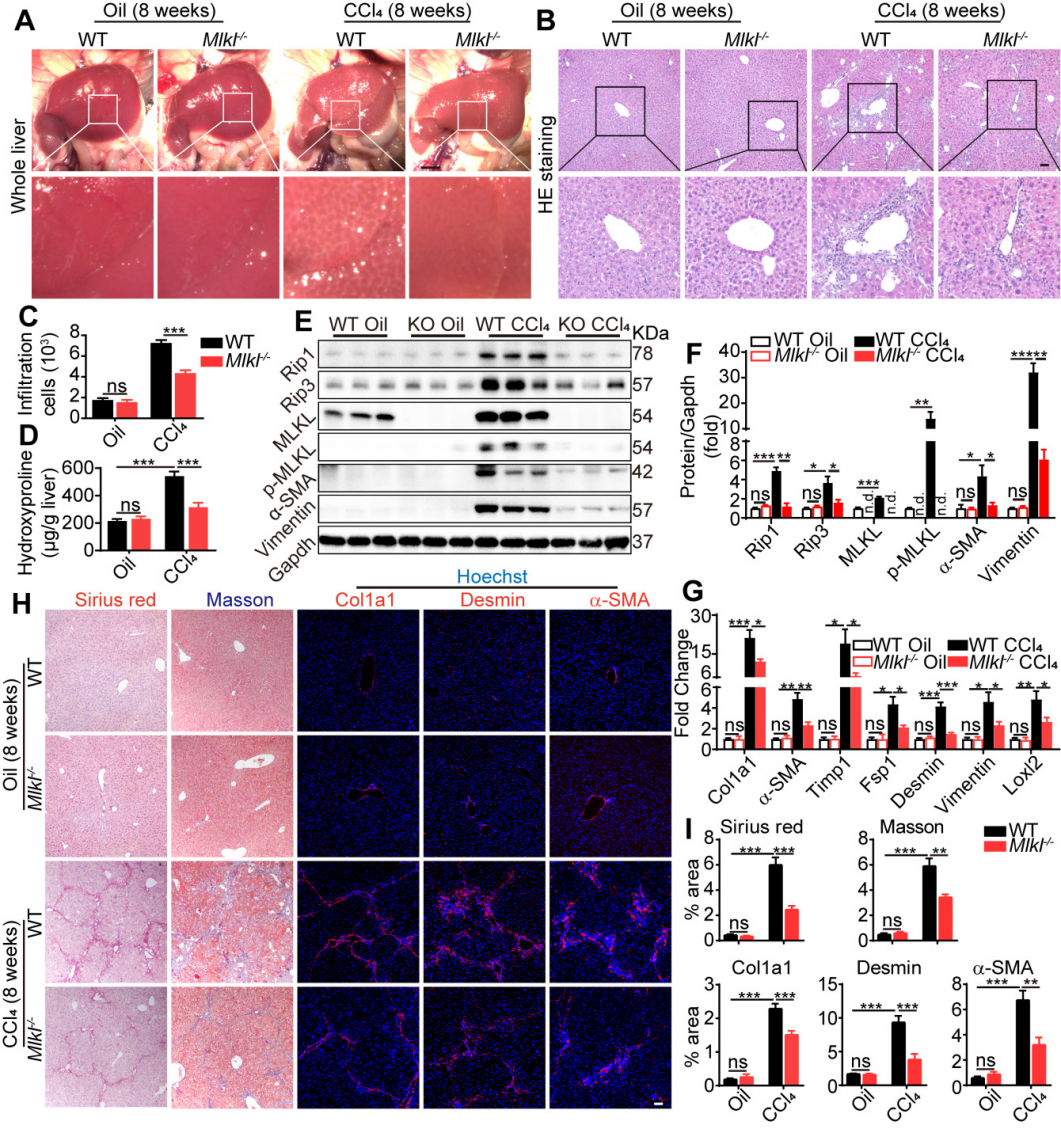
** Deletion of *Mlkl* attenuates hepatic fibrosis in chronic CCl_4_ model.** (A) Representative whole liver pictures of WT and *Mlkl*^-/-^ mice treated with oil or CCl_4_ (chronic). Scale bar represents 0.5 cm. (B) Representative H&E staining images of liver sections. (C) Quantification of the infiltrating cells in (B) (Oil groups, n=4; CCl_4_ groups, n=8). (D) Hydroxyproline levels in liver samples from WT and *Mlkl^-/-^* mice treated with oil or CCl_4_ (Oil groups, n=4; CCl_4_ groups, n=8). (E) Western blot analysis of MLKL, p-MLKL, Rip3, Rip1, and fibrosis markers α-SMA, Vimentin in the liver tissues (each lane represents one mouse). Gapdh was used as loading control. (F) Quantification of the blots in (E). All proteins were normalized to Gapdh in the same sample, then normalized to WT mice treated with vehicle (Oil). (G) Quantitative RT-PCR analysis of hepatic fibrosis genes *Col1a1*, *α-SMA*, *Timp1*, *Fsp1*, *Desmin*, *Vimentin*, and *Loxl2* in liver samples from WT and *Mlkl*^-/-^ mice treated with oil or CCl_4_ (Oil groups, n=4; CCl_4_ groups, n=8). (H) Representative images of Sirius red and Masson staining, and immunofluorescence staining of Col1a1, Desmin, and α-SMA in liver sections. Nuclei were stained with Hoechst 33342. (I) Quantitative analysis of positive staining areas in (H). (Oil groups, n=4; CCl_4_ groups, n=8). Data are shown as Means ± SEM, *P < 0.05, **P< 0.01, ***P< 0.001 (Student's t-test). Scale bar represents 100 µm.

Eight weeks after CCl_4_ treatment, livers were harvested and fibrotic changes could be clearly visualized in WT mice, while such changes were less visible in livers from *Mlkl^-/-^* mice (Figure 3A). Hematoxyin and eosin (H&E) staining of the liver sections revealed that the severe immune cell infiltration in the CCl_4_-treated WT group was significantly reduced in *Mlkl^-/-^* group (Figure 3B-C). Moreover, hydroxyproline was significantly increased in the liver of CCl_4_-treated WT mice, while the increase was significantly less in *Mlkl*^-/-^ mice (Figure 3D). Immunoblotting showed clear upregulation of proteins involved in necroptosis, including MLKL/p-MLKL, RIP1 and RIP3, while deletion of MLKL significantly reduced these essential components in necroptosis, together with fibrotic proteins, including Vimentin and α-SMA, after CCl_4_ treatment (Figure 3E-F). More fibrotic genes were analyzed by quantitative RT-PCR, and the results showed that the mRNA levels of* Col1a1*, *a-SMA, Timp1, Fsp1, Desmin, Vimentin,* and *Loxl2* were much higher in the CCl_4_-WT livers than those in the CCl_4_-*Mlkl^-/-^* livers (Figure 3G). Sirius Red staining and Masson staining both revealed that the fibrotic areas were significantly reduced in CCl_4_-*Mlkl^-/-^* livers than in CCl_4_-WT livers (Figure 3H-I). Immunofluorescence staining also revealed reduced levels of Col1a1, Desmin, and α-SMA staining in the CCl_4_-*Mlkl^-/-^* livers, indicating less fibrotic changes in these animals (Figure 3H-I). Taken together, these data suggest that knockout of *Mlkl* in mice attenuates CCl_4_-induced hepatic fibrosis.

### Knockout of *Mlkl* in mice reduces BDL-induced liver fibrosis

**Figure 4:**
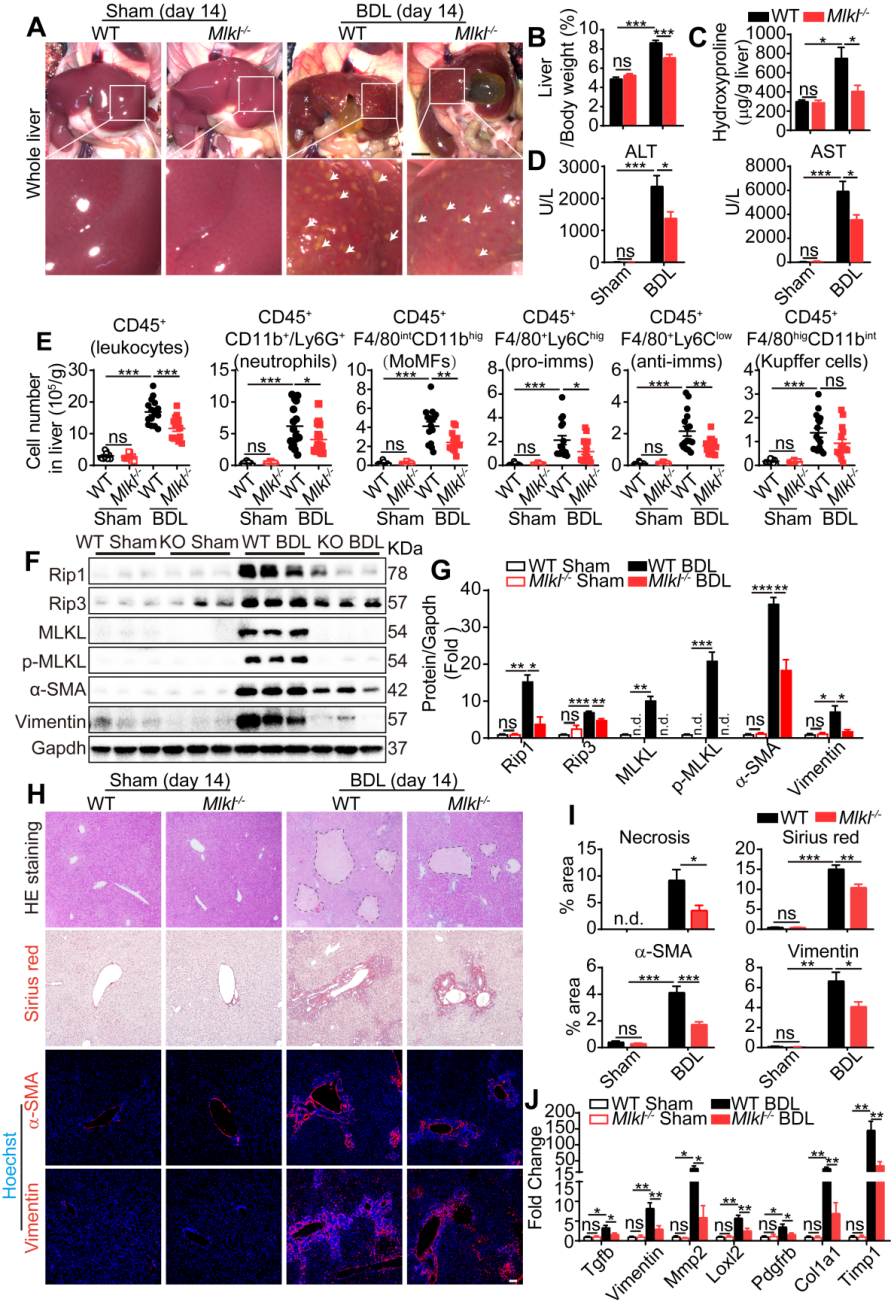
** Knockout of *Mlkl* attenuates BDL-induced liver injury.** (A) Representative pictures of whole liver from WT or *Mlkl*^-/-^ mice, 14 days after BDL, sham-operated mice were used as control. Bile infarcts (arrows) on the liver surface were indicated. Scale bar represents 0.5 cm. (B) Quantification of the liver/body weight ratio in (A) (Sham groups, n=10; BDL groups, n=16). (C) Hydroxyproline levels in the livers of sham or BDL mice (day 14) (Sham groups, n=10; BDL groups, n=16). (D) The amounts of ALT and AST in the serum of sham or BDL mice (day 14) (sham groups, n=10; BDL groups, n=16). (E) Quantification of the numbers of total leukocytes, neutrophils, MoMFs, pro-imms, anti-imms, and Kupffer cells, in the livers of sham or BDL mice (day 14) (sham groups, n=10; BDL groups, n=16). (F) Western blot analyses of Rip1, Rip3, MLKL, p-MLKL, α-SMA, and Vimentin in sham and BDL mice liver samples (each lane represents one animal). (G) Quantification of the blots in (F). All proteins were normalized to Gapdh in the same sample, then normalized to WT-sham mice. (H) Representative images of H&E staining, Sirius red staining, and immunofluorescence staining of α-SMA and Vimentin on Paraffin-embedded liver sections. Nuclei were stained with Hoechst 33342. (I) Quantification of positive staining areas in (H) (sham groups, n=3; WT-BDL, n=6, *Mlkl*^-/-^-BDL, n=7). (J) Quantitative RT-PCR analysis of hepatic fibrosis genes *Tgfb, Vimentin, Mmp2, Loxl2, Pdgfrb, Col1a1*,* and Timp1* in liver samples (sham groups, n=3; WT-BDL, n=6, *Mlkl*^-/-^-BDL, n=7). Data are shown as Means ± SEM, *P < 0.05, **P< 0.01, ***P< 0.001 (Student's t-test). Scale bar represents 100 µm.

Cholestatic liver diseases such as primary biliary cholangitis (PBC) and primary sclerosing cholangitis (PSC) result in chronic cholestasis which will further progress to hepatitis and liver fibrosis ultimately. Bile duct ligation (BDL) is a classical mouse model to study cholestasis induced liver injury. Fourteen days after BDL, WT and *Mlkl^-/-^* mice were sacrificed and the macroscopic necrotic foci of bile infarcts were clearly visible in BDL-WT livers, but much less obvious in BDL-*Mlkl^-/-^* livers (Figure 4A). The liver/body weight ratio was significantly increased in BDL-WT group versus Sham-WT group, while the ratio was significantly lower in BDL-*Mlkl^-/-^* group (Figure 4B). Hydroxyproline was significantly increased in the liver of BDL-WT mice, while BDL-*Mlkl*^-/-^ mice showed much less increase (Figure 4C). Moreover, the BDL-*Mlkl^-/-^* mice had significantly lower ALT and AST levels in serum, indicating less liver damage (Figure 4D). BDL led to the infiltration of large amount of immune cells into the liver (Figure 4E), however, the numbers of total infiltrating leukocytes, neutrophils, MoMFs, pro-imms, and anti-imms were significantly reduced in the BDL-*Mlkl^-/-^* group, compared to the BDL-WT group (Figure 4E). BDL also led to a significant increase in the protein levels of RIP1, RIP3, MLKL, p-MLKL, α-SMA, and Vimentin in the livers of WT mice, while *Mlkl* deletion significantly reduced the necroptotic proteins and the fibrosis markers in the liver (Figure 4F-G). H&E staining revealed that the BDL-induced necrotic areas in *Mlkl^-/-^* mice were significantly reduced compared to WT mice (Figure 4H-I). Sirius red staining and immunofluorescence staining of α-SMA and Vimentin all revealed that the fibrotic areas were significantly reduced in BDL-*Mlkl^-/-^* livers compared to BDL-WT livers (Figure 4H-I). Quantitative RT-PCR results also showed a significant reduction of fibrosis-related genes, including *Tgfb, Vimentin, Mmp2, Loxl2, Pdgfrb, Col1a1, and Timp1* in the livers of BDL-*Mlkl^-/-^* mice compared to that of BDL-WT mice (Figure 4J). Collectively, these results demonstrate that MLKL contributes to the initiation and progression of liver fibrosis.

### *Mlkl* deletion does not affect macrophage differentiation and function *in vitro*

Liver fibrosis is a multicellular response to liver injury. It is generally believed that hepatocyte damage causes inflammatory responses, and then the pro-inflammatory cells produce cytokines to activate and transdifferentiate quiescent hepatic stellate cells into myofibroblasts, leading to extracellular matrix deposit and progressive liver fibrosis 32, 33. We found that MLKL is ubiquitously expressed in isolated primary liver parenchyma cells (hepatocytes), CD11b^+^ cells, and HSCs (Figure S3A-B). MLKL was also detectable in human liver cell line (HepG2), human myeloid leukemia mononuclear cell line (Thp-1), and human hepatic stellate cell line (LX2) (Figure S3C-D).

Macrophage is one of the most abundant cell types in CD11b^+^ cells. Previous studies have shown that liver injury triggers monocytes migration from bone marrow to the site of injury, then monocytes polarize into either pro-inflammatory or anti-inflammatory macrophages 31, 34. To examine the role of MLKL in macrophage polarization, WT and *Mlkl^-/-^* bone marrow-derived macrophages (BMDMs, M0) were stimulated to induce classical (pro-inflammatory, M1) or alternative (anti-inflammatory and prohealing, M2) polarization. Both the WT-M0 and *Mlkl*^-/-^-M0 cells could be differentiated into typical M1 or M2 morphology (Figure S4A). Quantitative RT-PCR analysis showed the expression levels of M1 markers *Tnfa, IL-6, iNOS, IL-1β* and *IL-12,* and M2 markers *Ym1, Arg1, Mmr,* and *Fizz1* were similar in WT and *Mlkl*^-/-^ cells (Figure S4B). Immunofluorescence staining of iNOS and Cd206 in M0, M1, or M2 cells indicated increased levels of these proteins, but no differences were observed between WT and *Mlkl*^-/-^ cells (Figure S4C-D). Moreover, the production of pro-inflammatory cytokines IL-6, IL-1β, TNF-α, IFN-γ, and chemokine CCL2 were not significantly different between WT and *Mlkl*^-/-^ cells either (Figure S4E). These data indicate that knockout of *Mlkl* does not seem to affect macrophage polarization and function.

### *Mlkl* deletion attenuates CCl_4_ and bile acid induced hepatocytes injury *in vitro*

**Figure 5:**
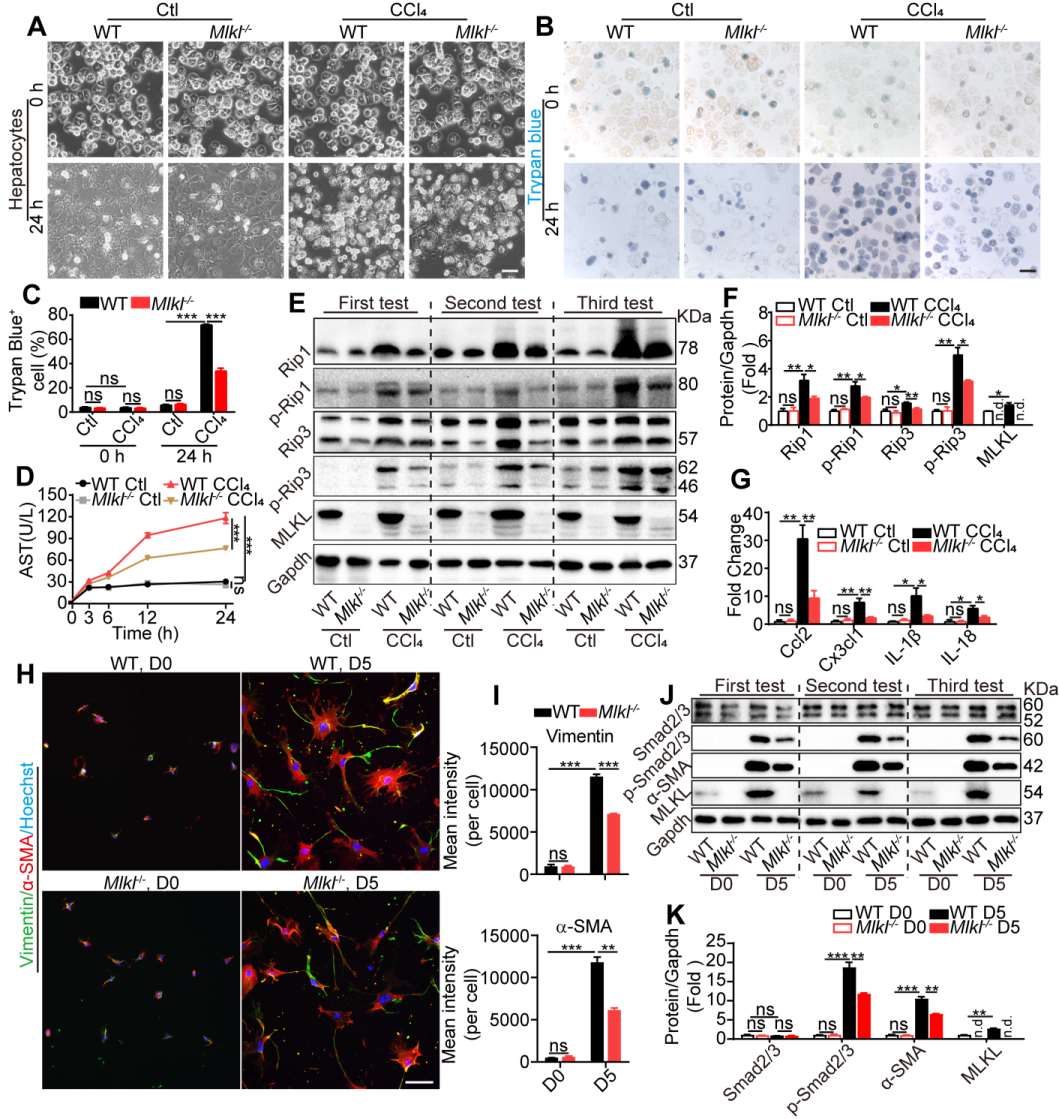
***Mlkl* deletion reduces hepatocyte necroptosis and HSC activation *in vitro***. (A) Representative morphology of WT and *Mlkl*^-/-^ hepatocytes treated with CCl_4_ or vehicle for 24 h. (B, C) Representative images (B) and statistics (C) of trypan blue staining in WT and *Mlkl*^-/-^ hepatocytes treated with CCl_4_ or vehicle for 24 h (n=3). (D) AST in the culture medium of WT and *Mlkl*^-/-^ hepatocytes treated with CCl_4_ or vehicle at the indicated time points (n=3). ***P< 0.001 (two-way ANOVA). (E) Western blot analyses of Rip1, p-Rip1, Rip3, p-Rip3, and MLKL in WT and *Mlkl*^-/-^ hepatocytes treated with CCl_4_ or vehicle for 24 h. Three independent experiments were shown. (F) Quantification of the blots in (E). All protein levels were first normalized to Gapdh in the same sample and then normalized to WT cells treated with vehicle. (G) qRT-PCR analysis of the mRNA level of *Ccl2*,* Cx3cl1*,* IL-1β* and* IL-18* in hepatocytes treated with CCl_4_ or vehicle for 24 h. (H) Immunofluorescence staining of α-SMA and Vimentin in HSCs freshly isolated from mice (D0) or cultured for 5 days (D5). (I) Quantification of mean fluorescence intensity in (H) (3 animals for each group, six random fields for each animal). (J) Western blot analyses of Smad2/3, p-Smad2/3, α-SMA, and MLKL in WT-HSCs and *Mlkl*^-/-^-HSCs at D0 and D5. Three independent experiments were shown. (K) Quantification of the blots in (J). All protein levels were first normalized to Gapdh in the same sample and then normalized to WT cells in D0. Data are shown as Means ± SEM, *P < 0.05, **P< 0.01, ***P< 0.001 (Student's t-test for C, F, G, I and K) . Scale bar represents 100 µm.

MLKL has been widely acknowledged to regulate cell necroptosis through phosphorylation and oligomerization at the plasma membrane 21. Hepatotoxicity is the initial cause of liver injury and hepatic fibrosis 18. We hypothesized that *Mlkl*^-/-^-hepatocytes may exhibit different responses to hepatotoxin-induced injury. *Mlkl*^-/-^- and WT-hepatocytes were exposed to CCl_4_ (10 mM) for 24 h to induce cell damage. WT-hepatocytes displayed obvious cell death, while *Mlkl*^-/-^-hepatocytes showed significantly less damage (Figure 5A). Trypan blue staining and Annexin V/PI staining showed that the ratio of positive (dead) cells in CCl_4_-*Mlkl*^-/-^-hepatocytes was significantly reduced comparing to the WT hepatocytes (Figure 5B-C, Figure S5A-B). After CCl_4_ treatment, the AST level in the culture medium was dramatically increased over time, but this phenomenon was significantly suppressed in *Mlkl*^-/-^-hepatocytes versus WT-hepatocytes (Figure 5D). Glycochenodeoxycholic acid (GCDC) is one of the important components in bile acid which could also induce liver and hepatocyte damage. *Mlkl* deficiency also protected against GCDC-induced hepatocyte injury (Figure S5C-D).

These observations indicate that necroptosis may be involved in hepatotoxin-induced hepatocyte damage. After CCl_4_ treatment, the protein levels of the essential components in necroptosis, including Rip1, p-Rip1, Rip3, p-Rip3, and MLKL, all dramatically increased. Interestingly, all these proteins were significantly reduced in CCl_4_-*Mlkl*^-/-^-hepatocytes versus CCl_4_-WT-hepatocytes (Figure 5E-F). Others have reported that injured hepatocytes would release chemokines CCL2, CX3CL1, and pro-inflammatory IL-1β, IL-18 which promote the recruitment of inflammatory cells to the liver 35, 36. We also found the expression of *Ccl2*, *Cxc3cl1*, *IL-1β*, and *IL-18* was increased in all CCl_4_-treated groups, but significantly less in CCl_4_-*Mlkl*^-/-^-hepatocytes (Figure 5G). These results may explain why the numbers of total infiltrating leukocytes were dramatically reduced in the liver of *Mlkl*^-/-^ mice treated with CCl_4_ or BDL.

Previously studies indicated that MLKL is the downstream of RIP1 and RIP3, but our results showed that MLKL may feedback and regulate RIP1 and RIP3 in hepatotoxin-induced hepatocyte damage. To further confirm these results, *Mlkl* in HepG2 cells was knocked down using shRNA (sh*Mlkl*) (Figure S6A-B) and the cells were treated with CCl_4_ or vehicle for 24 h (Figure S6C). *Mlkl* knockdown significantly reduced AST secretion induced by CCl_4_ treatment (Figure S6D). Western blot analysis revealed that RIP1, p-RIP1, RIP3, p-RIP3, MLKL, and p-MLKL were dramatically increased after CCl_4_ treatment, but were significantly reduced in CCl_4_-sh*Mlkl* group (Figure S6E). These results reveal that *Mlkl* deficiency protects mouse and human hepatocytes from hepatotoxin induced necroptosis.

### *Mlkl* deletion reduces HSCs activation *in vitro*

Both the mRNA and protein of MLKL could be detected in high levels in mouse HSCs and LX2, a human HSC line (Figure S3). HSC activation is a key step leading to liver fibrosis. Previous reports have revealed that HSCs cultured *in vitro* will become activated with morphology changes 37. We observed almost no morphology and retinoid storage differences between WT-HSCs and *Mlkl*^-/-^-HSCs in the quiescent state (day 0) (Figure S7A). After being cultured *in vitro* for 5 days, HSCs underwent a characteristic fibrotic phenotype change to differentiate into myofibroblasts (Figure S7B) and became activated to express fibrotic markers α-SMA and Vimentin. Interestingly, *Mlkl*^-/-^-HSCs showed significantly less staining for α-SMA and Vimentin (Figure 5H-I). TGF-β/Smad mediated signaling is critical for HSC activation and differentiation into myofibroblasts 38. *In vitro* culture for 5 days led to increased protein levels of MLKL and α-SMA, more interestingly, although the total Smad2/3 level did not change, the p-Smad2/3 was significantly increased (Figure 5J-K). Deletion of *Mlkl* reduced p-Smad2/3 and α-SMA levels (Figure 5J-K), indicating MLKL may promote the activation of TGF-β/Smad pathway. In LX-2 cells, knockdown of *Mlkl* with shRNA also led to significantly reduced levels of p-Smad2/3 and α-SMA (Figure S8). These results suggest a critical role of MLKL in HSCs activation by regulating the activation of TGF-β/Smad pathway.

### AAV-shRNA mediated specific knockdown of *Mlkl* in hepatocytes ameliorates CCl_4_ induced liver fibrosis

The above data demonstrated that MLKL participates in liver fibrosis by regulating hepatocyte death and HSC activation. Considering hepatocyte damage is the first step in liver fibrosis, we generated adeno-associated virus (AAV) type 8 carrying *Mlkl* shRNA (AAV8-sh*Mlkl*) or scramble shRNA (AAV8-scramble) under the control of the hepatocyte-specific thyroid hormone-binding globulin (TBG) promoter. The AAV also contained a GFP sequence controlled by the TBG promoter. Eight weeks after tail vein injection of AAV8-sh*Mlkl* or AAV8-scramble, GFP signal could be detected throughout the liver, but not in other tissues, and the knockdown efficiency was confirmed by quantitative RT-PCR (Figure S9A-B). Meanwhile, hepatocytes, immune cells and HSCs were isolated from the mice receiving tail vain injection of AAV. It was found that AAV-sh*Mlkl* could specifically knockdown MLKL in hepatocytes without affecting the immune cells and HSCs (Figure S9C). Immunofluorescence staining of liver sections also showed that GFP was almost exclusively co-localized with hepatocyte marker HNF4α, but not macrophage marker F4/80, cholangiocyte marker CK19, or HSC marker α-SMA (Figure S9D). These data indicate the specific delivery of shRNAs to the hepatocytes.

**Figure 6:**
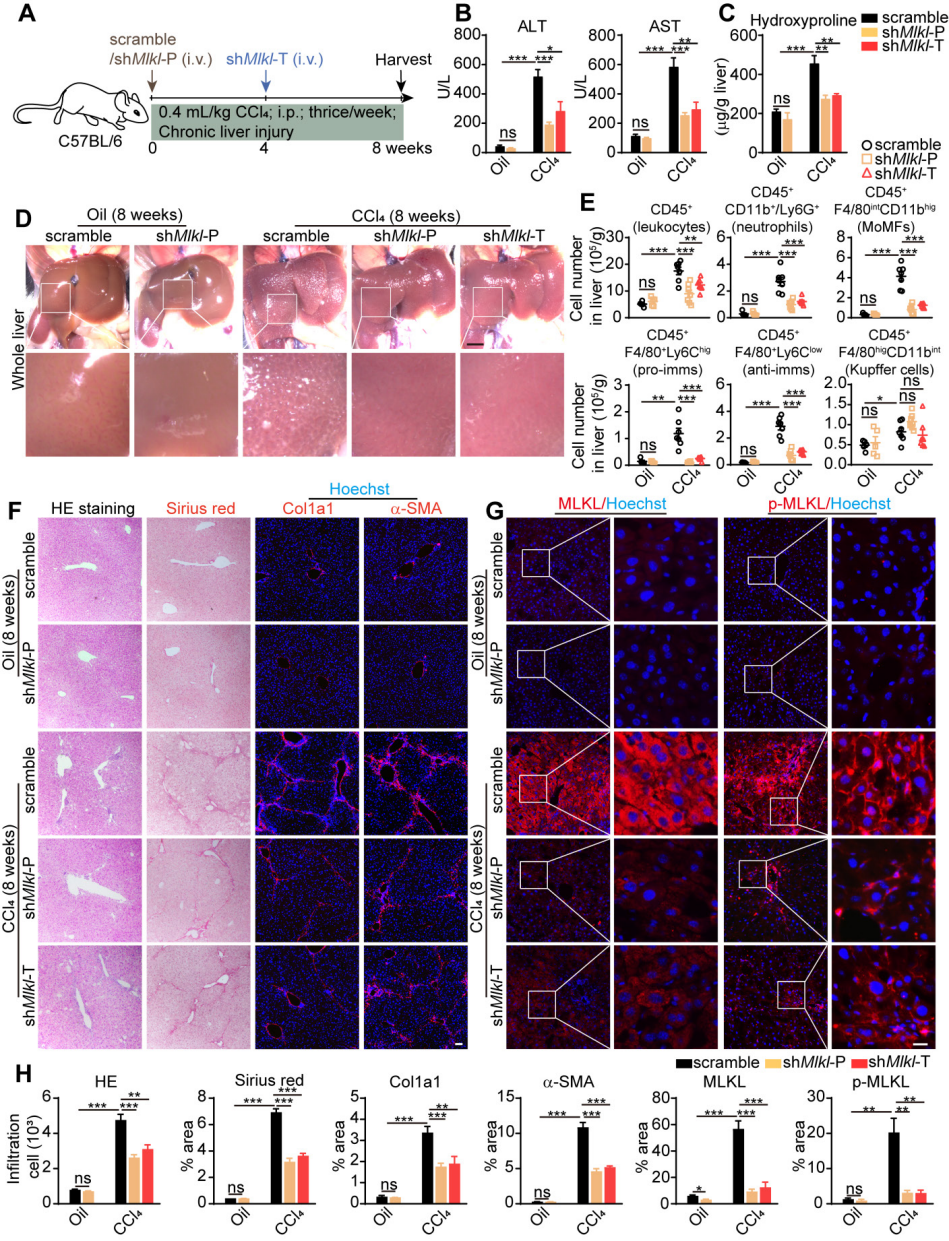
** AAV-shRNA mediated specific knockdown of *Mlkl* in hepatocytes reduces CCl_4_ induced liver fibrosis.** (A) Schematic of the experimental design of AAV treatment in CCl_4_-induced fibrosis in mice. AAV-sh*Mlkl* was given in either preventative (sh*Mlkl*-P) or therapeutic (sh*Mlkl*-T) manner. (B) Serum ALT and AST in mice received AAV-sh*Mlkl* and CCl_4_ (Oil groups, n=5; CCl_4_ groups, n=7). (C) Hydroxyproline levels in the livers of mice received AAV-sh*Mlkl* and CCl_4_ (Oil groups, n=5; CCl_4_ groups, n=7). (D) Representative whole liver pictures of mice received AAV-sh*Mlkl* (or scramble sequence) and CCl_4_ (or vehicle)-treatment. Scale bar represents 0.5 cm. (E) Quantification of the numbers of total leukocytes, neutrophils, MoMFs, pro-imms, anti-imms, and Kupffer cells in the liver of mice received AAV-sh*Mlkl* and CCl_4_. (Oil groups, n=5; CCl_4_ groups, n=7). (F) Representative images of H&E and Sirius red staining, and immunofluorescence staining of Col1a1, α-SMA on liver sections. Nuclei were stained with Hoechst 33342. (G) Representative images of immunofluorescence staining of MLKL or p-MLKL on liver tissue frozen sections. (H) Quantitative analysis of infiltration cells and positive staining areas in (F and G) (Oil groups, n=5; CCl_4_ groups, n=7. Data are shown as Means ± SEM, *P < 0.05, **P < 0.01, ***P< 0.001 (Student's t-test). Scale bar represents 100 µm.

AAV8-sh*Mlkl* was given both in prophylactic (sh*Mlkl*-P) and therapeutic (sh*Mlkl*-T) ways, and 8 weeks after CCl_4_ treatment, mice were sacrificed (Figure 6A). As expected, the ALT and AST levels in serum were increased in all CCl_4_-treated groups, but significantly less in both sh*Mlkl*-P and sh*Mlkl*-T groups (Figure 6B). Hydroxyproline was also increased in all CCl_4_-treated groups, but both sh*Mlkl*-P and sh*Mlkl*-T treatment significantly prevented such increase (Figure 6C). Visual inspection of the whole liver indicated less pathological changes in sh*Mlkl*-P and sh*Mlkl*-T mice treated with CCl_4_ (Figure 6D). FACS results also demonstrated that the number of total infiltrating leukocytes, neutrophils, MoMFs, pro-imms, or anti-imms were all significantly reduced in sh*Mlkl*-P and sh*Mlkl*-T groups versus scramble group (Figure 6E). H&E staining also showed less inflammatory cell infiltration (Figure 6F-H). Sirius red staining and immunofluorescence staining of Col1a1 and α-SMA revealed that the fibrotic areas were markedly reduced in both the sh*Mlkl*-P and sh*Mlkl*-T groups versus scramble group after CCl_4_-treatment (Figure 6F-H). Upregulation of MLKL and p-MLKL in hepatocytes after CCl_4_ treatment was further confirmed by immunostaining (Figure 6G-H), and as previously reported, the p-MLKL was located on the plasma membrane. Sh*Mlkl*-P and sh*Mlkl*-T treatment significantly reduced both MLKL and p-MLKL levels (Figure 6G-H). Western blotting also revealed that the increase in MLKL protein level after CCl_4_-treatment was significantly reduced by sh*Mlkl*-P and sh*Mlkl*-T injection, accompanied by the significant reduction of the fibrosis markers Vimentin and α-SMA (Figure S10A-B). The reduction of fibrosis-related genes after sh*Mlkl*-P and sh*Mlkl*-T was also confirmed by quantitative RT-PCR (Figure S10C). Taken together, these data demonstrate that knockdown of *Mlkl* with AAV-shRNA in hepatocytes markedly ameliorates CCl_4_-induced hepatocyte damage, liver inflammation, and hepatic fibrosis.

## Disscusion

Although many classes of pharmacological agents have been studied in clinical trials for the treatment of liver fibrosis 39, 40, there are no approved therapies to date. The strategies include removing the cause of liver injury based on various aetology, reducing hepatocyte damage, inhibiting hepatic inflammation, inhibiting myofibroblast activation or removal of hepatic myofibroblasts, modulating ECM deposition, etc. To a certain extent, liver fibrosis is a reversible process. There are clinical evidences from patients with chronic liver disease of diverse aetiology who have been successfully treated 41. In a clinical study with 5 years of treatment with the antiviral drug tenofovir in chronic hepatitis B patients, 74% of the patients showed extensive histological regression and were no longer considered to be cirrhotic 42, indicating liver fibrosis is a highly dynamic and bidirectional process and the removal of the cause of liver injury could initiate recovery.

In the real world, the initial cause of liver injury apart from viral infection might be more complicated. Thus protecting hepatocytes from dying would be the earlist step to stop the fibrosis process. A number of FXR or PPARγ agonists have been shown to reduce ALT levels in NASH or NAFLD patients, indicating hepatocytes protecting effects 39, 43. Emricasan, a pan caspase inhibitor, has been studied in clinical trials in NASH patients with stage 2 or 3 fibrosis or stage 1 fibrosis with risks for progression 43, with primary outcome being improvement in fibrosis without worsening NASH. However, the application of pan caspase inhibitor might be problematic, since inhibiting caspase 3 and 8 may reduce apoptosis, but will also unleash the caspase 8-mediated suppression of necroptosis 20, another type of programmed cell death which has also been found in liver injury in both patients and model animals.

Activated (phosphorylated) MLKL has been found in liver samples from patients with drug-induced liver injury 44, autoimmune hepatitis 45, and NASH 46. In animal models, previous studies have shown that RIP1 kinase activity promotes steatohepatitis by mediating macrophage cell death and inflammation 24. Ablation of *Rip3* prevents ethanol-induced liver injury and CCl_4_-induced liver fibrosis 25, 26, however, additional results have reported that RIP3 protects mice from HFD-induced liver injury 27. Deficiency of *Mlkl* alleviates hepatic insulin resistance and glucose intolerance 28, and has a protective effect on NASH induced by high FFC through inhibition of hepatocyte autophagy 29. Our data clearly show that the MLKL level has strong positive correlations with many fibrotic markers in patient liver samples. And genetic deletion of *mlkl* protects hepatocytes from hepatotoxin-induced necroptosis *in vitro* and reduces liver injury and fibrosis *in vivo*.

Another interesting finding in our study is that *mlkl^-/-^* directly reduces the activation of HSCs, possibly via the regulation of TGFβ/Smad 2/3 pathway. HSCs are activated mainly through TGF-β/Smad pathway and differentiate into myofibroblasts that express α-SMA and Vimentin, which are also EMT (epithelial-mesenchymal transition) markers 47, 48. Although it remains controversial whether the activation of HSC can be regarded as a classical EMT process 49, HSC in activation might possess some characteristics of the cells in the EMT process 50, 51. A previous study has demonstrated that depletion of *Mlkl* inhibits invasion of carcinoma cells by suppressing EMT 52. MLKL has also been reported to regulate mitochondrial respiration 53, and mitochondrial activity is increased when HSCs activate 54. MLKL can also form ion channels after activation 55. Ion channel such as TRPM7 participates in HSC activation 56, 57. Although our results indicate that MLKL may regulate HSC activation via TGFβ/Smad 2/3 pathway, the exact mechanism remains to be elucidated.

To our surprise, our data indicate that MLKL plays limited roles in macrophage polarization and functions. Activation of MLKL has been reported to induce macrophage necroptosis, and targeting macrophage necroptosis has been proposed to have therapeutic and diagnostic value for plaques in atherosclerosis 58. One recent publication has reported that upon LPS challenge, BMDMs from *Mlkl^-/-^* mice express lower *Tnf-α*, *IL-1β*, and *Mcp1* compared with WT cells 59. Our *in vitro* polarization experiment demonstrated clearly that *Mlkl* deletion did not influence BMDM polarization and cytokines production. Thus the reduced macrophages observed in *Mlkl^-/-^* liver after CCl_4_ or BDL treatment is largely due to reduced hepatocyte injury in* Mlkl^-/-^* mice, and thus less recruitment of immune cells.

Although from our data and previous reports, MLKL-mediated necroptosis plays a deteriorating role in liver injury and fibrosis, the pathway may have a positive impact on other tissues. For example, myofiber necroptosis promotes muscle stem cell proliferation during regeneration 60. Remyelination is driven by necroptosis of pro-inflammatory microglia and subsequent repopulation to a regenerative state 61. Although necroptosis has been reported to promote tumorigenesis, necroptosis also contributes to the death of cancer cells and the activation of anti-tumor immunity 62. Pharmacological inhibition of necroptosis promotes human breast cancer cell proliferation and metastasis 63, and some compounds that induce necroptosis have been reported to eliminate tumor cells 64. Thus, targeting this pathway for the treatment of liver fibrosis needs to be tissue- or cell-specific. AAVs in combination with cell specific promoters are the leading platform for *in vivo* delivery of gene therapies in tissue- or cell-specific way 65. Several AAV-based therapies have already been approved 66-68, and more are under clinical evaluation 65. Our data indicate that AAV8 mediated hepatocyte-specific *Mlkl* knockdown is an effective way in treating hepatic fibrosis.

In conclusion, MLKL plays a critical role in liver injury and fibrosis in both patients and animal models. *Mlkl* deletion in mice significantly reduces clinical symptoms of CCl_4_- and bile duct ligation (BDL)-induced liver injury and fibrosis. *Mlkl^-/-^* blocks liver fibrosis largely by reducing hepatocyte necroptosis and HSC activation. AAV8-mediated specific knockdown of *Mlkl* in hepatocytes remarkably alleviates CCl_4_-induced liver fibrosis in both preventative and therapeutic ways. Our results demonstrate targeting MLKL in hepatocytes might be an effective way to treat liver fibrosis.

## Materials and Methods

### Patients

Liver specimens from donors were obtained from Zhongshan Hospital (Fudan University, Shanghai, China) with the patient informed consent, which were immediately frozen and stored in liquid nitrogen until further analysis. All human subject studies were approved by the human ethics committees of the Zhongshan Hospital and were conducted in accordance with the principles of the Declaration of Helsinki. All patient samples were routinely processed for diagnosis and evaluated by pathologists in Zhongshan hospital. Patient information is shown in Table S1.

### Mice

All mice were housed under controlled humidity and temperature conditions and under 12-hour light/dark cycles. The methods were completed in line with the approved guidelines. All surgeries were performed using appropriate anesthesia, in compliance with the guidelines and ethics of the Institutional Animal Care and Use Committee (ACUC), Shanghai Institute of Materia Medica, Chinese Academy of Sciences. *Mlkl* knockout mice (*Mlkl^-/-^*) were generated with CRISPR-Cas9 technology. We intended to introduce a InDel between 1-80 bp in the exon 1 of *Mlkl* gene, which may lead to early stop of the translation. We identified three PAM sequences (XGG) in this region, so three sgRNAs adjacent to the PAM sequences were designed. Eventually, sgRNA (5'-TTGGGACAGATCATCAAGTT-3') was chosen since it led to high cutting efficiency in a cell-based reporter assay (data not shown). The sgRNA and Cas9 protein (Invitrogen, A36499) were mixed and microinjected into the zygotes to generate knockout mice. A founder mouse with 10 bp deletion starting from the 27th base of the transcription starting site in exon1 in one allele, which leads to a frameshift and incorrect translation from 9th amino acid and premature translation stop at 17th amino acid, was used to breed *Mlkl^-/-^* mice. Homozygous *Mlkl^-/-^* mice were born from heterozygote mating. Age- and gender-matched WT littermate mice served as controls and are referred to as WT mice. The genotypes of newborn mice were determined by PCR using tail tips with primers listed in Table S2.

### CCl_4_ induced liver injury

Mice at the age of 8-10 weeks were subjected to intraperitoneal (i.p.) injection of 0.6 mL/kg body weight CCl_4_ diluted in olive oil (1:9, vol:vol) or olive oil alone every other day for three times (acute injury). Or the mice were injected i.p. with 0.4 mL/kg body weight CCl_4_ diluted in olive oil, three times a week for eight weeks (chronic injury). After the last injection for 48 h, all mice were sacrificed via anesthesia overdose, blood taken for routine serum biochemistry. Livers were processed for qRT-PCR, Western blot, histological analysis, and flow cytometry analysis following established protocols.

### BDL model

The BDL operation was performed as previously reported 69. After anesthesia and midline laparotomy, the bile duct was exposed and underwent double ligature of the proximal common bile duct. For sham operation group, skin incision was done without bile-duct ligation. BDL mice developed cholestasis and associated fibrosis over a fourteen day period. At the end of the experiment, the mice were sacrificed, liver and body weight recorded. The blood samples and the liver specimens were harvested for subsequent experiments.

### Isolation of liver non-parenchymal cells (NPCs) and flow cytometric analysis

Liver NPCs were isolated according to previously reported methods with minor modifications 70. Briefly, the homogenate of the liver after perfusion was shaken at 37 °C for 30 min in a digestion solution (RPMI 1640 (Gibco) containing 25 µg/mL DNaseI (Roche) and 1 mg/mL collagenase IV (Gibco)). The cells were filtered through a 40 μm cell mesh and then erythrocytes were lysed using red blood cell lysis buffer for 5 min. After centrifugation for 5 min at 500 g, NPCs in the pellet were resuspended in PBS. CD45^+^ (Thermofisher; 8802-6865-74) and CD11b^+^ (Milteny Biotec; 130-049-601) cells in NPCs were enriched with magnetic bead sorting according to the manufacturer's instructions. The sorting efficiency was then verified by flow cytometry.

Subsequently, multicolor staining was conducted for analysis of the leukocytes in the liver, using combinations of the following mAbs: FITC-conjugated anti-CD45 Ab (invitrogen; 11-0451-82), APC-conjugated anti-F4/80 Ab (invitrogen; 17-4801-82), PECy7-conjugated anti-CD11b Ab (invitrogen; 25-0112-82), APCCy7-conjugated anti-Ly6G Ab (BD; 560600), PE-conjugated anti-Ly6C Ab (invitrogen; 12-5932-82). The cells were stained with fluorescence-conjugated surface antibodies for 30 min at 4 °C, washed three times with PBS, and then analyzed using a flow cytometer (ACEA Novocyte). Data were analyzed using NovoExpress software. Cells were defined as following: neutrophils (CD45^+^CD11b^+^Ly6G^+^), MoMFs (CD45^+^F4/80^int^CD11b^hig^), pro-inflammatory macrophages (pro-imms, CD45^+^F4/80^int^CD11b^hig^Ly6C^hig^), anti-inflammatory macrophages (anti-imms, CD45^+^F4/80^int^CD11b^hig^Ly6C^low^), and Kupffer cells (CD45^+^F4/80^hig^CD11b^int^).

### Primary hepatocyte isolation

Mouse hepatocytes were isolated using a standard 2-step perfusion technique. In brief, the liver was perfused with 15 mL of perfusion buffer (NaCl 8000 mg/L, KCl 400 mg/L, NaH_2_PO_4_ 600 mg/L, Na_2_HPO_4_ 600 mg/L, NaHCO_3_ 350 mg/L, EGTA 190 mg/L, Glucose 900 mg/L, pH 7.35-7.4) through the inferior vena at a flow rate of 3 mL/min, followed by 25 mL Enzyme buffer (NaCl 8000 mg/L, KCl 400 mg/L, NaH_2_PO_4_ 600 mg/L, Na_2_HPO_4_ 600 mg/L, NaHCO_3_ 350 mg/L, CaCl_2_·2H_2_O 560 mg/L, HEPES 190 mg/L, Collagenase Type 1 (Gibco) 600 mg/L, pH 7.35-7.4) at the same flow rate. After perfusion, the liver was removed from the abdominal cavity and hepatocytes were released into the M199 medium using sterile surgical scissors. The cell suspension was filtered through a 70 μm cell strainer (Corning). After centrifugation, hepatocytes were purified by 50% of percoll (Sigma) gradient at low-speed centrifugation (1,500 rpm, 15 min) then the pellets were dissociated into a single-cell suspension. The viability of isolated hepatocytes was about 90% as determined by Trypan blue staining. Finally, hepatocytes were seeded into gelatin-coated 24 well plates and cultured in M199 medium supplemented with 10% FBS and 1% penicillin-streptomycin.

### Mouse hepatic stellate cell (HSC) isolation

HSCs were isolated from mouse liver as previously reported 71. In brief, the liver was first perfused through the inferior vena with 10 mL of perfusate solution Ⅰ (NaCl 8000 mg/L, KCl 400 mg/L, NaH_2_PO4·H_2_O 88.17 mg/L, Na_2_HPO_4_ 120.45 mg/L, HEPES 2380 mg/L, NaHCO_3_ 350 mg/L, EGTA 190 mg/L, glucose 900 mg/L, pH 7.35-7.4). Then the liver was perfused with 25 mL of enzyme buffer (NaCl 8000 mg/L, KCl 400 mg/L, NaH_2_PO_4_·H_2_O 88.17 mg/L, Na_2_HPO_4_ 120.45 mg/L, HEPES 2380 mg/L, NaHCO_3_ 350 mg/L, CaCl_2_·2H_2_O 560 mg/L, pH 7.35-7.4) containing 10 mg pronase (Sigma-Aldrich). Finally, the liver was perfused with 35 mL of enzyme buffer containing 20 mg collagenase IV (Gibco). Immediately after perfusion, the liver cells were dissociated and suspended in 25 mL enzyme buffer containing 12.5 mg pronase and 15 mg collagenase IV, digested at 37 °C for 15 min. After pronase-collagenase perfusion and digestion, HSCs were obtained using discontinuous density gradient centrifugation as confirmed by retinoid autofluorescency. HSCs were seeded into gelatin-coated 24 well plates and cultured in DMEM supplemented with 1% FBS and 1% penicillin-streptomycin.

### Mouse bone marrow-derived macrophages (BMDMs) isolation and polarization

BMDMs were isolated from mouse tibias and femurs for the macrophage differentiation assay. Briefly, after isolation of the femur and tibia, the marrow cavities were rinsed three times using a 1-mL syringe. Red blood cell lysis buffer was used to remove the erythrocytes. BMDMs were incubated in DMEM with 10% FBS and macrophage colony stimulating factor (MCSF) 25 ng/mL (R&D system; 416-ML-050) for 7 days to differentiate into M0 macrophages. Fresh medium was changed every other day. On day 8, the media was changed to DMEM containing MCSF 25 ng/mL, LPS 100 ng/mL (MCE; HY-D1056), and IFN-γ 20 ng/mL (R&D system; 485-MI-100) to stimulate cells to polarize into M1-like macrophages. DMEM containing MCSF 25 ng/mL, IL-4 20 ng/mL (R&D system; 404-ML-050) and IL-13 10 ng/mL (R&D system; 413-ML-050) was used to stimulate cells to polarize into M2-like macrophages. After 24 h, the cells and the supernatants were collected for further analysis.

### Cell lines and culture condition

HepG2 cells were maintained in DMEM, supplemented with 10% FBS, 1% penicillin-streptomycin, at 37 °C in 5% CO_2_. LX2 cells were maintained in DMEM, supplemented with 1% FBS, 1% penicillin-streptomycin, at 37 °C in 5% CO_2_.

### H&E, masson and sirius red staining

Liver samples were fixed in 4% paraformaldehyde (PFA), embedded in paraffin, and cut into 5 μm-thick sections. For H&E staining, sections were first stained with hematoxylin solution for 10 min, and then stained with eosin alcohol solution for 5 min. Finally, the sections were dehydrated and mounted with Permount Mounting Medium. For Masson's Trichrome staining, slides were immersed in Biebrich Scarle-Acid Fuchsin solution for 10 min, repeated washes in 0.2% acetic acid. Then they were put into Aniline blue for 10 s, repeated washes in 0.2% acetic acid, dehydrated and coverslipped. For Sirius red staining, slides were incubated in saturated aqueous picric acid for 1 h, followed by wash steps and immersed in hematoxylin solution for 10 min. After dehydration and coverslipped, the images were captured by a microscope, and the average of positive area per field was counted fields at 10x magnification using Image-J image analysis software.

### Immunofluorescence staining

Tissue slides were incubated in PBS containing 0.3% Triton X-100 and 5% BSA for 1 h. The slides were incubated with primary antibodies at 4 °C overnight. After a thorough wash, cells were incubated with the appropriate fluorescence-conjugated secondary antibodies (1:500) for 1 h at room temperature. Finally, the cell nuclei were stained with Hoechst 33342 solution for 30 min. Cells were fixed in 4% PFA at room temperature for 30 min, then follow the same staining protocol as tissue slides. Images were captured with an Olympus IX71 inverted fluorescent microscope and analyzed by professional image analysis software (Image J).

Antibodies used include α-SMA (1:200; Invitrogen MAI-06110), collagen1a1 (1:200; Arigo arg21965), Vimentin (1:200; Abcam ab8978), HNF4α (1:200; Abcam ab201460), CK19 (1:200; Abcam ab52625), F4/80 (1:200; Abcam ab6640), MLKL (1:200; Abgent AP14272B), p-MLKL (1:200; Abcam ab196436), iNOS (1:200; Abcam ab178945) and Cd206 (1:200; Abcam ab64693).

### Western blot

Liver tissues or cells were lysed and homogenized in RIPA buffer supplemented with a protease inhibitor cocktail (Roche). Total protein was quantified using the BCA Protein Assay Kit (Thermo Fisher). Then protein extracts were boiled at 100 °C for 10 min in sample buffer (50 mM Tris-HCl, 2% w/v SDS, 10% glycerol, 1% β-mercaptoethanol, 0.01% bromophenyl blue (pH 6.8)). Cell lysates were separated on SDS-PAGE and transferred to polyvinylidene difluoride membranes. The membranes were first incubated with blocking buffer (TBS with 0.05% Tween 20, 10% nonfat milk) for 1 h at room temperature and then incubated overnight at 4 °C in buffer containing primary antibody. After a thorough wash, cells were incubated with the appropriate HRP-conjugated secondary antibodies (1:10,000) for 1 h. Immunostaining was visualized using Amersham ECL Plus Western Blotting detection reagents (GE Healthcare) and ChemiDoc imaging system (Bio-Rad).

Antibodies used include human MLKL (1:1000; Abcam ab194699), human p-MLKL (1:1000; CST 91689S), mouse MLKL (1:1000; Abgent AP14272B), mouse p-MLKL (1:1000; Abcam ab196436), RIP1 (1:1000; CST 3493), p-RIP1 (1:1000; CST 31122), RIP3 (1:1000; Abcam ab152130), p-RIP3 (1:1000; CST 91702), α-SMA (1:1000; Invitrogen MA1-06110), Vimentin (1:1000; Abcam ab8978), Smad2/3 (1:1000; CST 8685), p-Smad2/3 (1:1000; CST 8828), Gapdh (1:10,000; CST 8884).

### Quantitative RT-PCR

Total mRNA was isolated using Trizol (Invitrogen) and 1 μg RNA was reversed to cDNA using a PrimeScriptTM RT reagent kit (Takara) according to the manufacturer's protocol. Real-time PCR was performed using Hieff Qpcr SYBR Green Master Mix (Yeasen) and analyzed with a Stratagene Mx 3000P thermal cycler. Primer sequences are supplied in Table S3.

### *Mlkl* knockdown in human cell lines

Short hairpin RNA (shRNA) targeting human *Mlkl* mRNA (Table S4) was cloned into the PLKO.1/U6 plasmid. HEK293T cells were co-transfected with PLKO.1 (lentiviral plasmid), psPAX2 (packaging plasmid), and pMD2.G (envelope plasmid). Supernatants were collected 48 h after transfection. Lentiviral particles were filtered and stored at -80 °C until use. HepG2 or LX2 cells were seeded in 24-well plates and grown to 70%∼80% confluence. Then the medium was replaced by virus-containing supernatant supplemented with 8 μg/mL polybrene (Millipore), and the plates were centrifuged at 2,500 rpm for 90 min to ensure viral infection. Knockdown efficiency was determined using quantitative RT-PCR and Western blot 96 h later.

### AAV8 treatment

AAV8 containing scramble or sh*Mlkl* sequences (Table S5) under the control of TBG promoter was produced by Obio Technology (Shanghai). For prophylactic treatment, a single dose of AAV8 (10^12^ genome copies/mouse) was delivered by tail vein 24 h before CCl_4_ injection which lasted for 8 weeks. For therapeutic treatment, a single dose of AAV8 was injected via the tail vein after 4 weeks of CCl_4_ injection, and the CCl_4_ injection lasted for another 4 weeks. 48 h after the last CCl_4_ injection, the mice were euthanized and sacrificed. The blood and liver tissues were harvested for further analyses.

### Blood chemistry, hydroxyproline, and cytokine measurement

ALT, AST were determined by commercial kits (BioAssay Systems) and a multimode plate reader. The amount of hydroxyproline was determined using a hydroxyproline assay kit (Sigma, MAK008) according to manufacturer's instructions. Quantification of IFN-γ, IL-1β, IL-6, and CCL2 in the culture supernatant and serum were determined using Homogeneous Time-Resolved Fluorescence assays (Cisbio) according to the manufacturer's instructions.

### Statistical analysis

Values are reported as the means ± SEM. Statistical differences between the two groups were analyzed with two-tailed Student's t-test. All correlation analyses were performed using Pearson's correlation. To compare the secretion of AST from two groups for a period of time, two-way ANOVA was performed in Figure 5D, Figure S5D, and Figure S6D. P values less than 0.05 were considered statistically significant. All graphs were plotted with GraphPad Prism software. The Immunofluorescence staining and positive cell percentage were analyzed using ImageJ software.

## Supplementary Material

Supplementary figures and tables.

## References

[1] Lee YA, Wallace MC, Friedman SL (2015). Pathobiology of liver fibrosis: a translational success story. Gut.

[2] Bataller R, Brenner DA (2005). Liver fibrosis. J Clin Invest.

[3] Troeger JS, Mederacke I, Gwak GY, Dapito DH, Mu XR, Hsu CC (2012). Deactivation of Hepatic Stellate Cells During Liver Fibrosis Resolution in Mice. Gastroenterology.

[4] Lo RC, Kim H (2017). Histopathological evaluation of liver fibrosis and cirrhosis regression. Clin Mol Hepatol.

[5] Pellicoro A, Ramachandran P, Iredale JP, Fallowfield JA (2014). Liver fibrosis and repair: immune regulation of wound healing in a solid organ. Nat Rev Immunol.

[6] Friedman SL, Neuschwander-Tetri BA, Rinella M, Sanyal AJ (2018). Mechanisms of NAFLD development and therapeutic strategies. Nat Med.

[7] Castera L, Friedrich-Rust M, Loomba R (2019). Noninvasive Assessment of Liver Disease in Patients With Nonalcoholic Fatty Liver Disease. Gastroenterology.

[8] Schwabe RF, Tabas I, Pajvani UB (2020). Mechanisms of Fibrosis Development in Nonalcoholic Steatohepatitis. Gastroenterology.

[9] Chawla A, Nguyen KD, Goh YP (2011). Macrophage-mediated inflammation in metabolic disease. Nat Rev Immunol.

[10] Kisseleva T, Brenner D (2021). Molecular and cellular mechanisms of liver fibrosis and its regression. Nat Rev Gastro Hepat.

[11] Mridha AR, Wree A, Robertson AAB, Yeh MM, Johnson CD, Van Rooyen DM (2017). NLRP3 inflammasome blockade reduces liver inflammation and fibrosis in experimental NASH in mice. J Hepatol.

[12] Tacke F (2017). Targeting hepatic macrophages to treat liver diseases. Journal of Hepatology.

[13] Krenkel O, Tacke F (2017). Liver macrophages in tissue homeostasis and disease. Nat Rev Immunol.

[14] Higashi T, Friedman SL, Hoshida Y (2017). Hepatic stellate cells as key target in liver fibrosis. Adv Drug Deliv Rev.

[15] Schuppan D, Surabattula R, Wang XY (2018). Determinants of fibrosis progression and regression in NASH. J Hepatol.

[16] Seki E, Schwabe RF (2015). Hepatic inflammation and fibrosis: functional links and key pathways. Hepatology.

[17] Luedde T, Kaplowitz N, Schwabe RF (2014). Cell death and cell death responses in liver disease: mechanisms and clinical relevance. Gastroenterology.

[18] Luedde T, Kaplowitz N, Schwabe RF (2014). Cell Death and Cell Death Responses in Liver Disease: Mechanisms and Clinical Relevance. Gastroenterology.

[19] Fritsch M, Gunther SD, Schwarzer R, Albert MC, Schorn F, Werthenbach JP (2019). Caspase-8 is the molecular switch for apoptosis, necroptosis and pyroptosis. Nature.

[20] Schwabe RF, Luedde T (2018). Apoptosis and necroptosis in the liver: a matter of life and death. Nat Rev Gastroenterol Hepatol.

[21] Sun LM, Wang HY, Wang ZG, He SD, Chen S, Liao DH (2012). Mixed Lineage Kinase Domain-like Protein Mediates Necrosis Signaling Downstream of RIP3 Kinase. Cell.

[22] Samson AL, Zhang Y, Geoghegan ND, Gavin XJ, Davies KA, Mlodzianoski MJ (2020). MLKL trafficking and accumulation at the plasma membrane control the kinetics and threshold for necroptosis. Nat Commun.

[23] Afonso MB, Rodrigues PM, Carvalho T, Caridade M, Borralho P, Cortez-Pinto H (2015). Necroptosis is a key pathogenic event in human and experimental murine models of non-alcoholic steatohepatitis. Clin Sci.

[24] Majdi A, Aoudjehane L, Ratziu V, Islam T, Afonso MB, Conti F (2020). Inhibition of receptor-interacting protein kinase 1 improves experimental non-alcoholic fatty liver disease. Journal of Hepatology.

[25] Roychowdhury S, McMullen MR, Pisano SG, Liu XL, Nagy LE (2013). Absence of Receptor Interacting Protein Kinase 3 Prevents Ethanol-Induced Liver Injury. Hepatology.

[26] Wei S, Zhou HM, Wang Q, Zhou S, Li CY, Liu R (2019). RIP3 deficiency alleviates liver fibrosis by inhibiting ROCK1-TLR4-NF-kappa B pathway in macrophages. Faseb J.

[27] Roychowdhury S, McCullough RL, Sanz-Garcia C, Saikia P, Alkhouri N, Matloob A (2016). Receptor interacting protein 3 protects mice from high-fat diet-induced liverinjury. Hepatology.

[28] Xu H, Du X, Liu G, Huang S, Du W, Zou S (2019). The pseudokinase MLKL regulates hepatic insulin sensitivity independently of inflammation. Mol Metab.

[29] Wu X, Poulsen KL, Sanz-Garcia C, Huang E, McMullen MR, Roychowdhury S (2020). MLKL-dependent signaling regulates autophagic flux in a murine model of non-alcohol-associated fatty liver and steatohepatitis. J Hepatol.

[30] Conos SA, Chen KW, De Nardo D, Hara H, Whitehead L, Nunez G (2017). Active MLKL triggers the NLRP3 inflammasome in a cell-intrinsic manner. Proc Natl Acad Sci U S A.

[31] Karlmark KR, Zimmermann HW, Roderburg C, Gassler N, Wasmuth HE, Luedde T (2010). The Fractalkine Receptor CX(3)CR1 Protects Against Liver Fibrosis by Controlling Differentiation and Survival of Infiltrating Hepatic Monocytes. Hepatology.

[32] Trautwein C, Friedman SL, Schuppan D, Pinzani M (2015). Hepatic fibrosis: Concept to treatment. J Hepatol.

[33] Mederacke I, Hsu CC, Troeger JS, Huebener P, Mu XR, Dapito DH (2013). Fate tracing reveals hepatic stellate cells as dominant contributors to liver fibrosis independent of its aetiology. Nat Commun.

[34] Ju C, Tacke F (2016). Hepatic macrophages in homeostasis and liver diseases: from pathogenesis to novel therapeutic strategies. Cell Mol Immunol.

[35] Marra F, Tacke F (2014). Roles for chemokines in liver disease. Gastroenterology.

[36] Szabo G, Petrasek J (2015). Inflammasome activation and function in liver disease. Nat Rev Gastroenterol Hepatol.

[37] Qu C, Zheng DD, Li S, Liu YJ, Lidofsky A, Holmes JA (2018). Tyrosine kinase SYK is a potential therapeutic target for liver fibrosis. Hepatology.

[38] Meng XM, Nikolic-Paterson DJ, Lan HY (2016). TGF-beta: the master regulator of fibrosis. Nat Rev Nephrol.

[39] Friedman SL, Neuschwander-Tetri BA, Rinella M, Sanyal AJ (2018). Mechanisms of NAFLD development and therapeutic strategies. Nat Med.

[40] Kisseleva T, Brenner D (2021). Molecular and cellular mechanisms of liver fibrosis and its regression. Nat Rev Gastroenterol Hepatol.

[41] Ellis EL, Mann DA (2012). Clinical evidence for the regression of liver fibrosis. J Hepatol.

[42] Marcellin P, Gane E, Buti M, Afdhal N, Sievert W, Jacobson IM (2013). Regression of cirrhosis during treatment with tenofovir disoproxil fumarate for chronic hepatitis B: a 5-year open-label follow-up study. The Lancet.

[43] Harrison SA, Goodman Z, Jabbar A, Vemulapalli R, Younes ZH, Freilich B (2020). A randomized, placebo-controlled trial of emricasan in patients with NASH and F1-F3 fibrosis. J Hepatol.

[44] Wang H, Sun L, Su L, Rizo J, Liu L, Wang LF (2014). Mixed lineage kinase domain-like protein MLKL causes necrotic membrane disruption upon phosphorylation by RIP3. Mol Cell.

[45] Dara L, Liu ZX, Kaplowitz N (2016). A murder mystery in the liver: who done it and how?. J Clin Invest.

[46] Gautheron J, Vucur M, Luedde T (2015). Necroptosis in Nonalcoholic Steatohepatitis. Cell Mol Gastroenterol Hepatol.

[47] Masola V, Carraro A, Granata S, Signorini L, Bellin G, Violi P (2019). *In vitro* effects of interleukin (IL)-1 beta inhibition on the epithelial-to-mesenchymal transition (EMT) of renal tubular and hepatic stellate cells. J Transl Med.

[48] Gough NR, Xiang X, Mishra L (2021). TGF-beta Signaling in Liver, Pancreas, and Gastrointestinal Diseases and Cancer. Gastroenterology.

[49] Dewidar B, Meyer C, Dooley S, Meindl-Beinker AN (2019). TGF-beta in Hepatic Stellate Cell Activation and Liver Fibrogenesis-Updated 2019. Cells.

[50] Fabregat I, Caballero-Diaz D (2018). Transforming Growth Factor-beta-Induced Cell Plasticity in Liver Fibrosis and Hepatocarcinogenesis. Front Oncol.

[51] Tsuchida T, Friedman SL (2017). Mechanisms of hepatic stellate cell activation. Nat Rev Gastroenterol Hepatol.

[52] Dong Y, Sun Y, Huang Y, Fang X, Sun P, Dwarakanath B (2019). Depletion of MLKL inhibits invasion of radioresistant nasopharyngeal carcinoma cells by suppressing epithelial-mesenchymal transition. Ann Transl Med.

[53] Majdi A, Aoudjehane L, Ratziu V, Islam T, Afonso MB, Conti F (2020). Inhibition of receptor-interacting protein kinase 1 improves experimental non-alcoholic fatty liver disease. J Hepatol.

[54] Trivedi P, Wang S, Friedman SL (2021). The Power of Plasticity-Metabolic Regulation of Hepatic Stellate Cells. Cell Metab.

[55] Xia B, Fang S, Chen X, Hu H, Chen P, Wang H (2016). MLKL forms cation channels. Cell Res.

[56] Fang L, Huang C, Meng X, Wu B, Ma T, Liu X (2014). TGF-β1-elevated TRPM7 channel regulates collagen expression in hepatic stellate cells via TGF-β1/Smad pathway. Toxicology and Applied Pharmacology.

[57] Zhu Y, Men R, Wen M, Hu X, Liu X, Yang L (2014). Blockage of TRPM7 channel induces hepatic stellate cell death through endoplasmic reticulum stress-mediated apoptosis. Life Sci.

[58] Karunakaran D, Geoffrion M, Wei LH, Gan W, Richards L, Shangari P (2016). Targeting macrophage necroptosis for therapeutic and diagnostic interventions in atherosclerosis. Sci Adv.

[59] Miyata T, Wu X, Fan X, Huang E, Sanz-Garcia C, Ross CKC (2021). Differential role of MLKL in alcohol-associated and non-alcohol-associated fatty liver diseases in mice and humans. JCI Insight.

[60] Zhou S, Zhang W, Cai G, Ding Y, Wei C, Li S (2020). Myofiber necroptosis promotes muscle stem cell proliferation via releasing Tenascin-C during regeneration. Cell Res.

[61] Lloyd AF, Davies CL, Holloway RK, Labrak Y, Ireland G, Carradori D (2019). Central nervous system regeneration is driven by microglia necroptosis and repopulation. Nat Neurosci.

[62] Zhu F, Zhang W, Yang T, He SD (2019). Complex roles of necroptosis in cancer. J Zhejiang Univ Sci B.

[63] Shen F, Pan X, Li M, Chen Y, Jiang Y, He J (2020). Pharmacological Inhibition of Necroptosis Promotes Human Breast Cancer Cell Proliferation and Metastasis. Onco Targets Ther.

[64] Fulda S (2014). Therapeutic exploitation of necroptosis for cancer therapy. Semin Cell Dev Biol.

[65] Wang D, Tai PWL, Gao G (2019). Adeno-associated virus vector as a platform for gene therapy delivery. Nat Rev Drug Discov.

[66] Yla-Herttuala S (2012). Endgame: glybera finally recommended for approval as the first gene therapy drug in the European union. Mol Ther.

[67] Darrow JJ (2019). Luxturna: FDA documents reveal the value of a costly gene therapy. Drug Discov Today.

[68] Iftikhar M, Frey J, Shohan MJ, Malek S, Mousa SA (2021). Current and emerging therapies for Duchenne muscular dystrophy and spinal muscular atrophy. Pharmacol Ther.

[69] Martin K, Pritchett J, Llewellyn J, Mullan AF, Athwal VS, Dobie R (2016). PAK proteins and YAP-1 signalling downstream of integrin beta-1 in myofibroblasts promote liver fibrosis. Nat Commun.

[70] Lu L, Woo J, Rao AS, Li YP, Watkins SC, Qian SG (1994). Propagation of Dendritic Cell Progenitors from Normal Mouse-Liver Using Granulocyte-Macrophage Colony-Stimulating Factor and Their Maturational Development in the Presence of Type-1 Collagen. J Exp Med.

[71] Mederacke I, Dapito DH, Affo S, Uchinami H, Schwabe RF (2015). High-yield and high-purity isolation of hepatic stellate cells from normal and fibrotic mouse livers. Nat Protoc.

