# Supplementary material for: Loss of MLKL ameliorates liver fibrosis by inhibiting hepatocyte necroptosis and hepatic stellate cell activation
Source: Theranostics. 2022 Jul 4;12(11):5220–36. doi: 10.7150/thno.71400 (PMC9274737; doi:10.7150/thno.71400)
Supplement: Supplementary file 1 — Supplementary figures and tables. [file thnov12p5220s1.pdf]

Supplementary Information

Loss of MLKL ameliorates liver fibrosis by inhibiting liver parenchymal cell necroptosis and hepatic stellate cell activation

Ren Guo[1,#], Xiaohui Jia[1,2,#], Zhenbin Ding[3,4,5], Gang Wang[6], Mengmeng Jiang[7], Bing Li[1,2,8], Shanshan Chen[1,2], Bingqing Xia[1], Qing Zhang[1,8], Jian Liu[1], Ruting Zheng[1,2,7], Zhaobing Gao[1,9], Xin Xie[1,2,7,8,9,*]

[1]State Key Laboratory of Drug Research, National Center for Drug Screening, Shanghai Institute of Materia Medica, Chinese Academy of Sciences, Shanghai 201203, China

[2]University of Chinese Academy of Sciences, No.19A Yuquan Road, Beijing 100049, China

[3]Department of Liver Surgery and Transplantation, Liver Cancer Institute, Zhongshan Hospital, Fudan University, Shanghai 200031, China

[4]Key Laboratory of Carcinogenesis and Cancer Invasion of Ministry of Education, Shanghai 200031, China

[5]Shanghai Xuhui Central Hospital, Zhongshan-Xuhui Hospital, Fudan University, Shanghai 200031, China

[6]Department of Pharmaceutics, School of Pharmacy, Fudan University, Shanghai 201203, China

[7]School of Life Science and Technology, ShanghaiTech University, Shanghai 201210, China

[8]School of Pharmaceutical Science and Technology, Hangzhou Institute for Advanced Study, University of Chinese Academy of Sciences, Hangzhou 310024, China

[9]CAS Key Laboratory of Receptor Research, Shanghai Institute of Materia Medica, Chinese Academy of Sciences, Shanghai 201203, China

Figure S1. Construction of *Mlkl* knockout mice and genotype identification

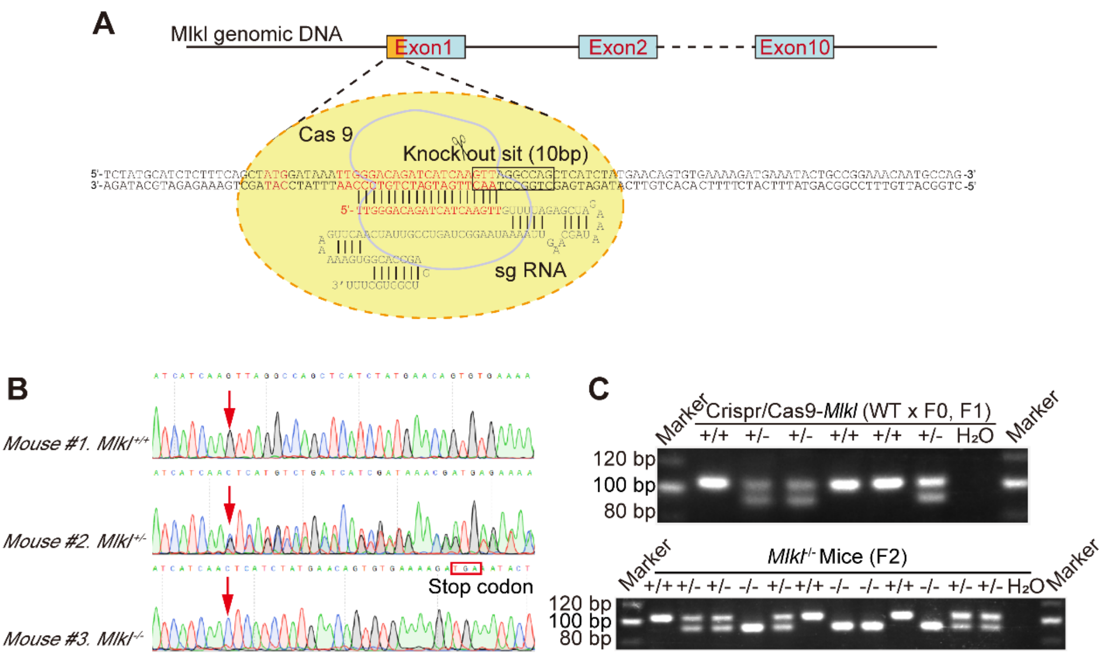

(A) Schematic diagram of *Mlkl* gene deletion with CRISPR-Cas9 system. (B) Sequencing chromatograms showing confirmed *Mlkl* knockout. Arrow indicated the first mutation base. Mouse #2 is the founder with 10 bp deletion starting from the 27th base of the transcription starting site in exon1 in one allele, leading to a premature translation stop at the 17th amino acid. (C) Typical PCR results for genomic identification of *Mlkl*$^{+/+}$, *Mlkl*$^{+/-}$, and *Mlkl*$^{-/-}$ mice.

Figure S2. *Mlkl* deletion reduces leukocytes accumulation in the liver after acute CCl_4 treatment

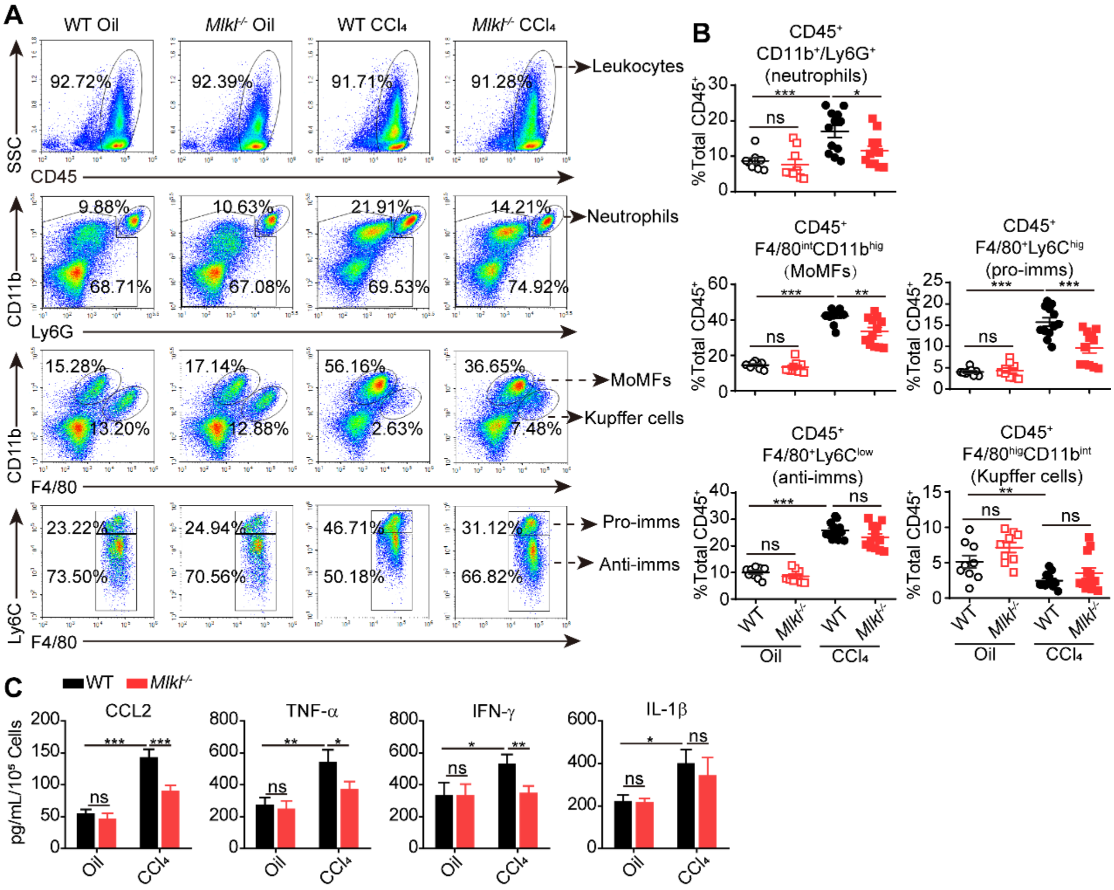

(A, B) Representative flow cytometric images (A) and the percentage (B) of neutrophils, MoMFs, pro-imms, anti-imms, and Kupffer cells within total $CD45^+$ cells in the liver of WT and *Mlkl*$^{-/-}$ mice treated with oil or CCl_4 (acute) (Oil groups, n=9; CCl_4 groups, n=12). (C) Levels of CCL2, TNF-α, IFN-γ, and IL-1β in the culture supernatants of $CD11b^+$ cells isolated from the livers of WT and *Mlkl*$^{-/-}$ mice treated with oil or CCl_4 (acute) (n=11). Data are shown as Means ± SEM, *P < 0.05, **P< 0.01, ***P< 0.001 (Student's t-test).

Figure S3. The expression of MLKL in liver parenchymal and nonparenchymal cells

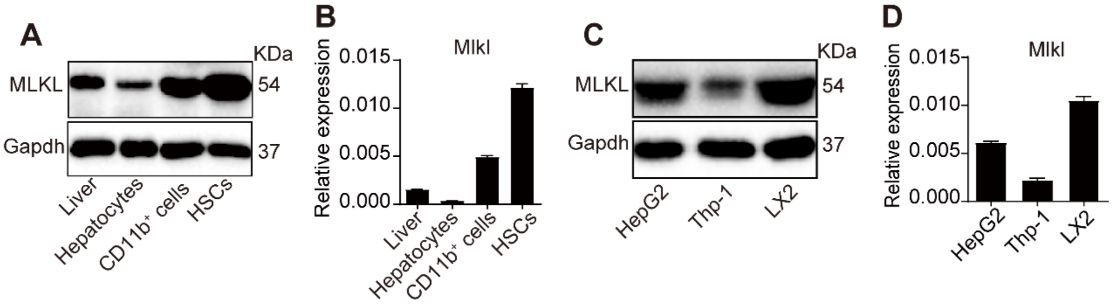

(A, B) Western blot (A) and quantitative RT-PCR (B) analysis of MLKL level in mouse hepatocytes, CD11b$^+$ cells, and HSCs. (C, D) Western blot (C) and quantitative RT-PCR (D) analysis of MLKL level in the human cell lines. All data are shown as Means ± SEM (n=3).

Figure S4. *Mlkl* deletion does not affect macrophage polarization and function

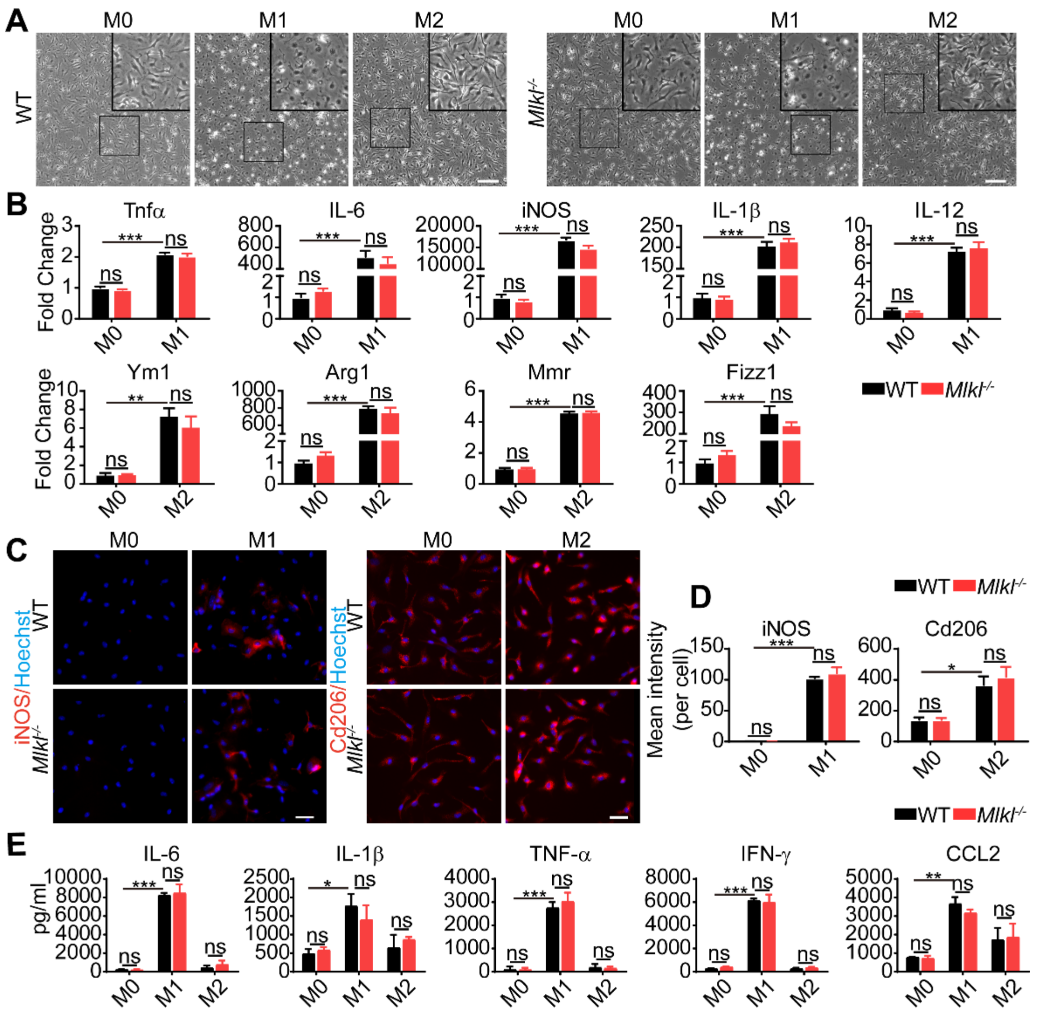

(A) Morphology of WT and *Mlkl*$^{-/-}$ monocyte-derived M0, M1, and M2 macrophages. (B) Quantitative RT-PCR analysis of the M1 macrophage markers including *Tnfα, IL-6, iNOS, IL-1β, IL-12, and* M2 macrophage markers including *Ym1, Arg1, Mmr, Fizz1* in WT and *Mlkl*$^{-/-}$ monocyte-derived macrophages (n=3). (C) Immunofluorescence staining of iNOS and Cd206 in macrophages. Nuclei were stained with Hoechst 33342. (D) Statistical data of the intensity of iNOS and Cd206 staining in (C) (n=3). (E) Levels of IL-6, IL-1β, TNF-α, IFN-γ, and CCL2 in the supernatants of M0, M1, and M2 culture. All data are shown as Means ± SEM, *P < 0.05, **P< 0.01, ***P< 0.001 (Student's t-test). Scale bar represents 100 μm.

Figure S5. Knockout of *Mlkl* attenuates hepatocytes death

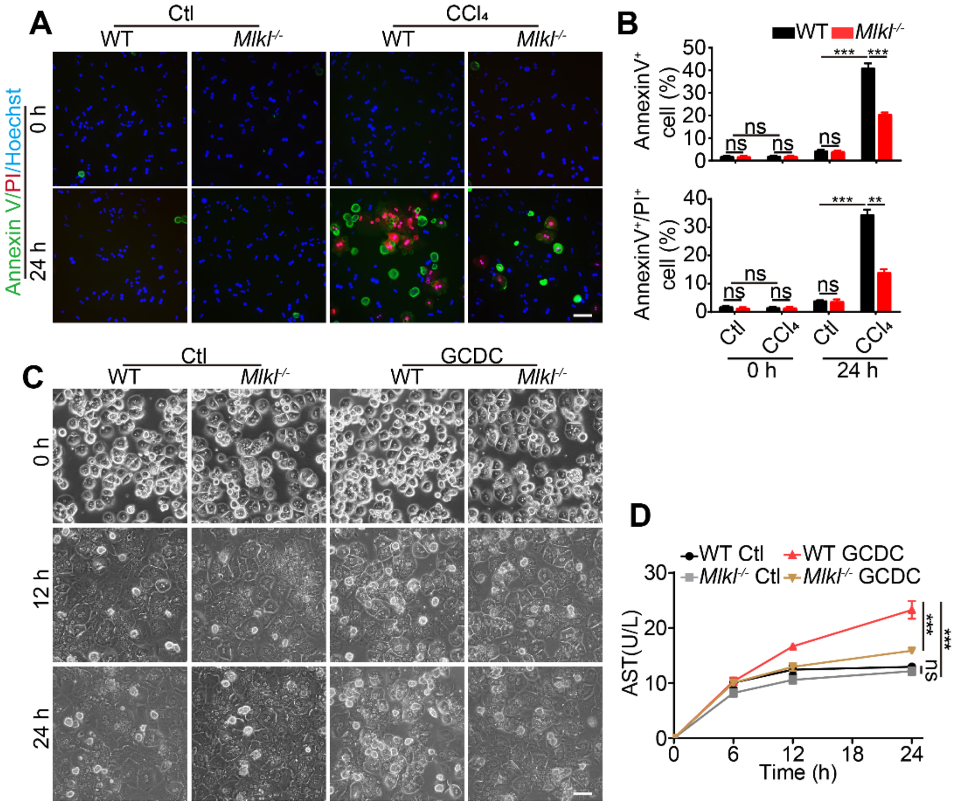

(A) Representative images of Annexin V and PI staining in WT and *Mlkl*$^{-/-}$ hepatocytes treated with vehicle or CCl_4 for 24 h. (B) Statistical analysis of Annexin V^+ and Annexin V^+/PI^+ cells in (A) (n=3). **P< 0.01, ***P< 0.001 (Student's t-test). (C) Representative morphology of WT and *Mlkl*$^{-/-}$ hepatocytes treated with GCDC (100 μM) or vehicle ctl for 12 and 24 h. (D) Measurement of AST in the culture medium of WT and *Mlkl*$^{-/-}$ hepatocytes treated with GCDC (100 μM) or vehicle ctl at the indicated time points (n=3). ***P< 0.001 (two-way ANOVA). All data are shown as Means ± SEM. Scale bar represents 100 μm.

Figure S6. Knockdown of *Mlkl* in HepG2 inhibits CCl_4 induced death

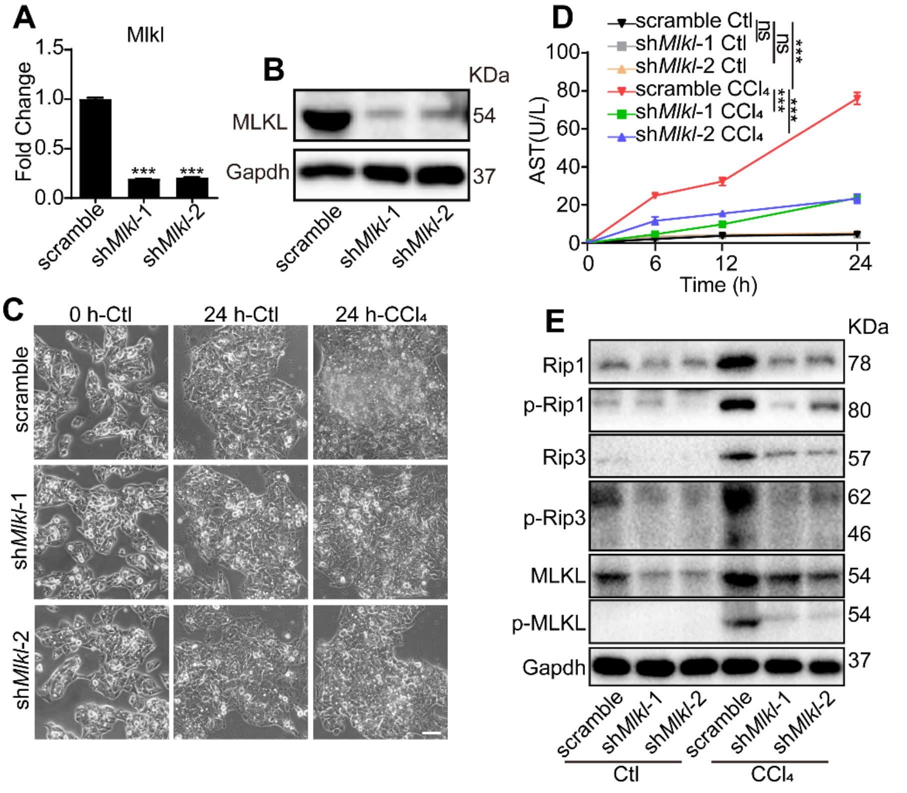

(A, B) Quantitative RT-PCR (A) and western blot (B) analysis of MLKL expression in HepG2 cells after transfected with sh*Mlkl* (or scramble sequence) for 96 h. (C) Representative morphology of HepG2 cells transfected with scramble, sh*Mlkl*-1, and sh*Mlkl*-2 for 96 h and then treated with CCl_4 or vehicle for 24 h. (D) AST level in the culture medium of WT and $Mlkl^{-/-}$ hepatocytes treated with CCl_4 or vehicle ctl at the indicated time points (n=3). Data are Means ± SEM, ***P< 0.001 (two-way ANOVA). (E) Western blot analysis of Rip1, p-Rip1, Rip3, p-Rip3, MLKL, and p-MLKL in HepG2 cells transfected with scramble, sh*Mlkl*-1, and sh*Mlkl*-2 and then treated with CCl_4 for 24 h. Gapdh was used as loading control. Scale bar represents 100 µm.

Figure S7. Knockout of *Mlkl* prevents HSC activation *in vitro*

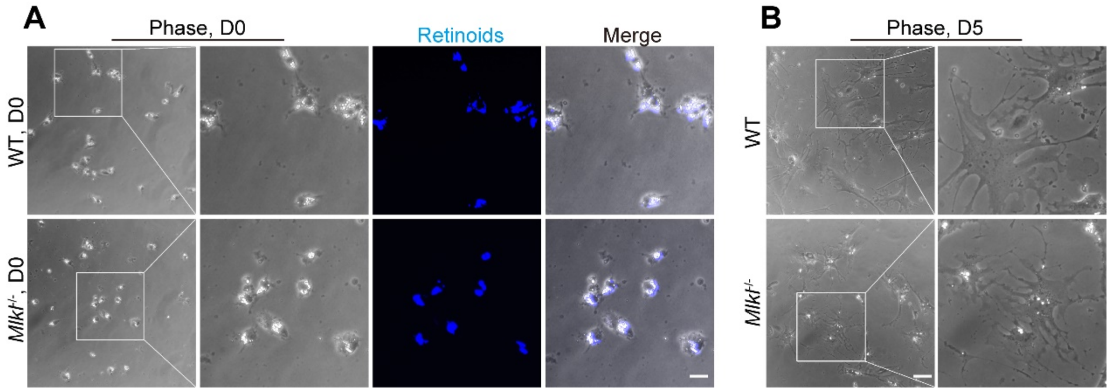

(A) Representative morphology and retinoids fluorescence of freshly isolated WT and *Mlkl*$^{-/-}$ HSCs. (B) Representative morphology of WT and *Mlkl*$^{-/-}$ HSCs cultured in vitro for 5 days. Scale bar represents 100 µm.

Figure S8. Knockdown of *Mlkl* represses LX2 activation and pro-fibrotic phenotype

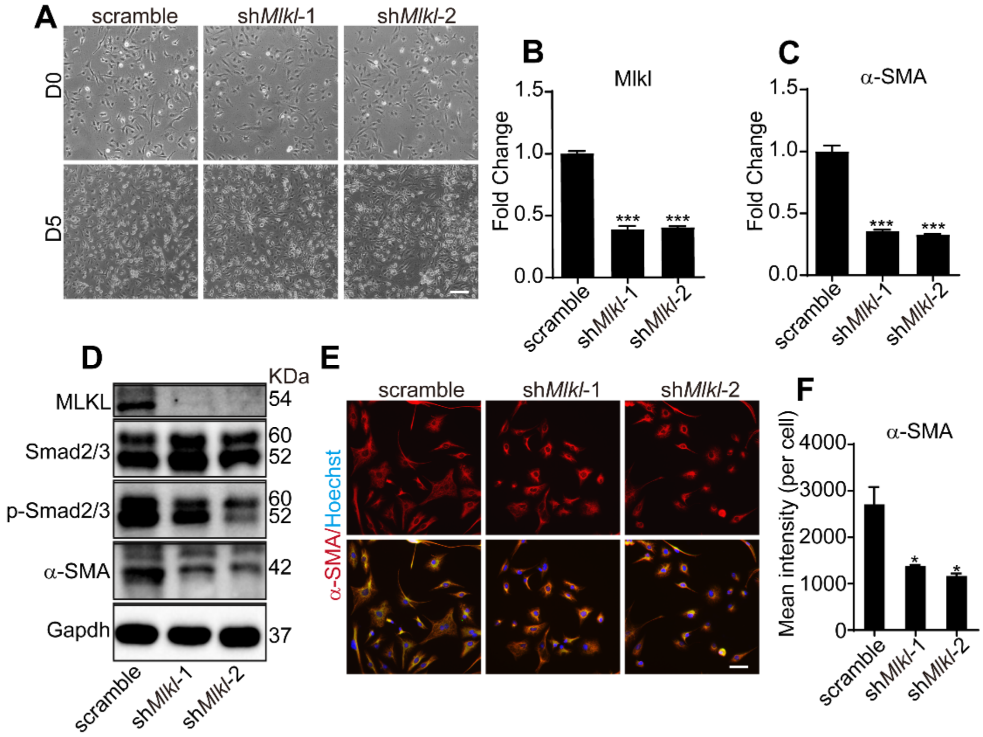

(A) Representative morphology of LX2 cells after transfected with sh*Mlkl* (or scramble) at day 0 and day 5. (B, C) Quantitative RT-PCR analysis of the *Mlkl* (B) and *α-SMA* (C) in shRNA transfected cells at day 5 (n=3). (D) Western blot analysis of MLKL, p-Smad2/3, and α-SMA in shRNA transfected cells (or scramble) at day 5. Gapdh was used as loading control. (E) Immunofluorescence staining of α-SMA in shRNA transfected cells at day 5. (F) The α-SMA fluorescence intensity in (E) was quantified using ImageJ software. Data are shown as Means ± SEM, *P < 0.05, ***P < 0.001 (Student's t-test). Scale bar represents 100 μm.

Figure S9. AAV8-TBG efficiently infects liver parenchymal cells

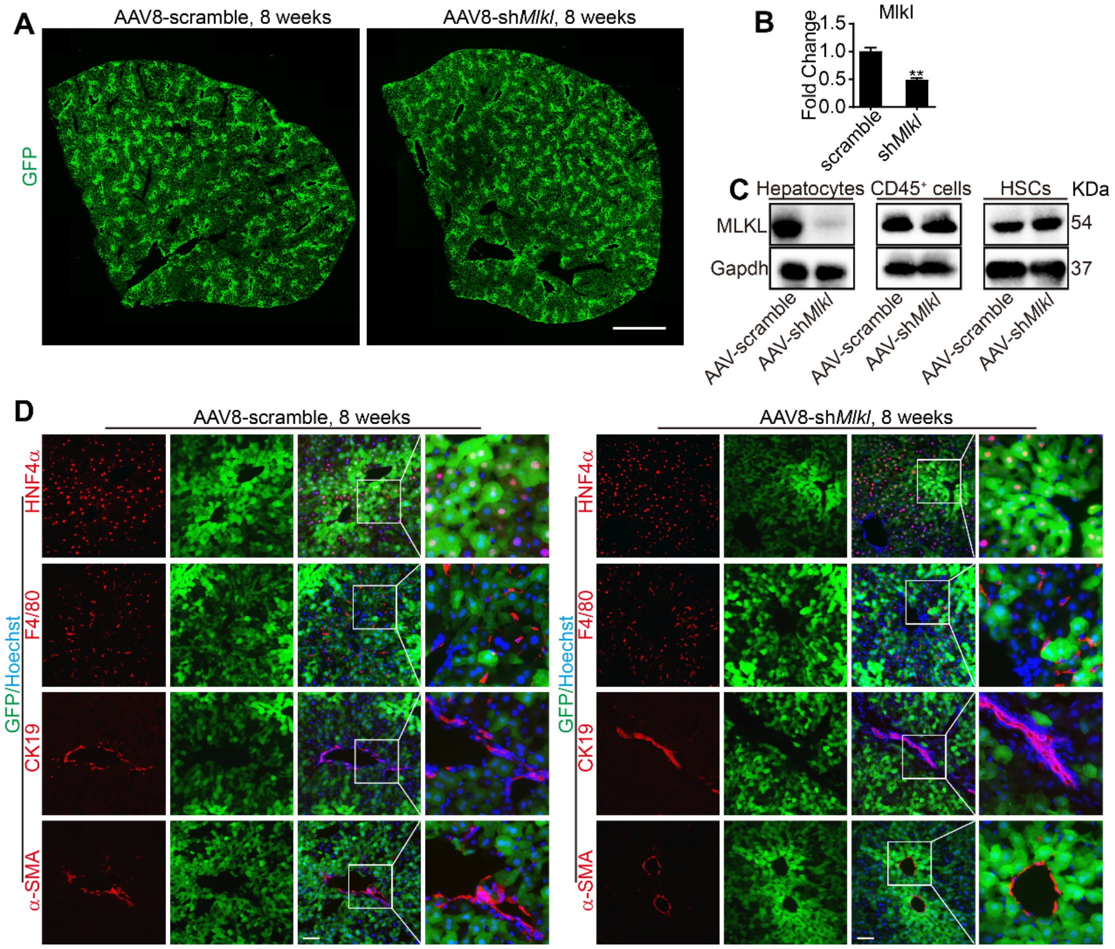

(A) The GFP fluorescence images of the frozen sections of the livers in mice 8 weeks after receiving tail vein injection of AAV8-scramble or AAV8-sh*Mlkl*. Scale bar represents 2 mm. (B) Quantitative RT-PCR analysis of *Mlkl* expression in the liver of mice 8 weeks after AAV injection (n=3). (C) Eight weeks after tail vein injection of AAV8-TBG-scramble or AAV8-TBG-sh*Mlkl*, hepatocytes, immune cells ($CD45^+$), and HSCs were isolated from liver tissues. The knockdown efficiency and specificity were analyzed by western blotting. (D) Co-localization of GFP with hepatocyte marker HNF4α, macrophage marker F4/80, cholangiocyte marker CK19, and HSC marker α-SMA in mice 8 weeks after AAV injection. Data are shown as Means ± SEM, *P < 0.05, ***P < 0.001 (Student's t-test). Scale bar represents 100 μm.

Figure S10. AAV-shRNA mediated specific knockdown of *Mlkl* in hepatocytes ameliorates CCl_4 induced liver fibrosis

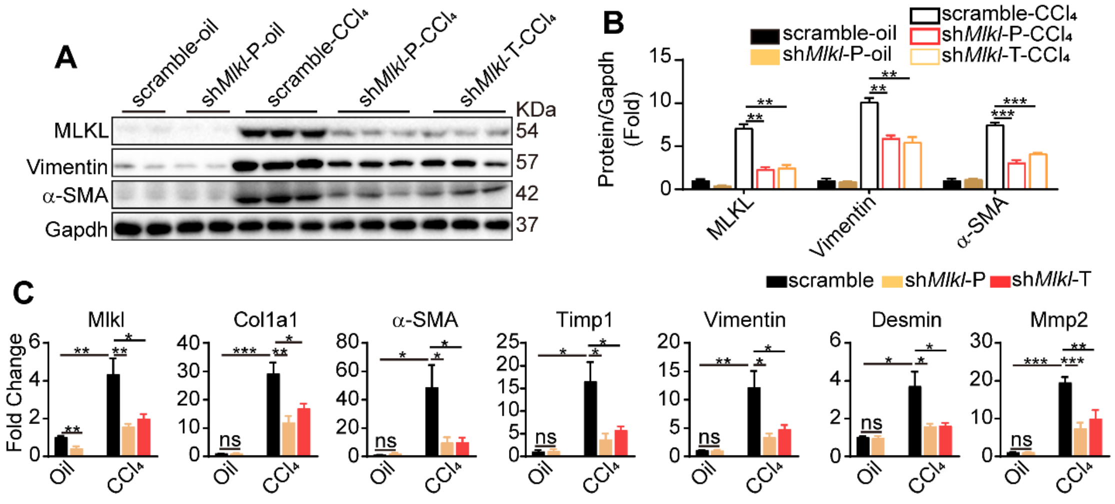

(A) Western blot analysis of MLKL, Vimentin, and α-SMA in liver samples of mice treated as Figure 6A (each lane represents one animal). (B) Quantification of the blots in (A). All proteins were normalized to Gapdh in the same sample, then normalized to mice receiving AAV-scramble and treated with vehicle (oil). (C) Quantitative RT-PCR analysis of *Mlkl* and hepatic fibrosis genes including *Col1a1, a-SMA, Timp1, Vimentin, Desmin, Fsp1*, and *Mmp2* in the liver samples (Oil groups, n=5; CCl_4 groups, n=7). Data are shown as Means ± SEM, *P < 0.05, **P < 0.01, ***P< 0.001 (Student's t-test).

Table S1. Patient information

Characteristics	Fibrosis Stage		
	F2	F3	F4
Patient Number	9	8	28
Gender(M/F)	7/2	5/3	25/3
Age(years)	60.00±3.50	58.63±4.02	59.57±2.02
ALT(U/L)	27.11±4.21	26.63±5.71	32.57±4.35
AST(U/L)	23.78±2.42	27.88±5.02	38.43±4.68
ALP(U/L)	73.33±5.72	70.13±7.79	105.00±12.09
GGT(IU/L)	55.67±14.03	34.63±6.60	89.89±25.69
TBIL(μmol/L)	12.69±1.86	14.74±1.06	19.74±3.93
Glucose(mmol/L)	6.13±0.59	5.68±0.54	5.40±0.30
Total cholesterol (mmol/L)	4.32±0.40	4.19±0.28	3.81±0.13
HDL cholesterol (mmol/L)	1.05±0.09	1.31±0.10	1.32±0.49
LDL cholesterol (mmol/L)	2.51±0.35	2.40±0.24	2.02±0.12
Triglycerides(mmol/L）	1.78±0.45	1.16±0.21	1.06±0.13

Note: M/F, male/female; F2, F3, or F4, different fibrosis stage of human liver diseases; ALT, alanine aminotransferase; AST, aspartate aminotransferase; ALP, alkaline phosphatase; GGT, glutamyl transpeptidase; TBIL, total bilirubin; Data are shown as Means ± SEM.

Table S2. Mlkl Genotyping Primers

Name	Sequence (5'-3')
Mlkl-Forward	CATCTCTTTCAGCTATGGATAAATT
Mlkl-Reverse	GCTGGCATTGTTTCCGGCAGTA

Table S3. Primer pairs for RT-qPCR Analysis of Gene Expression

Gene	Forward (5'-3')	Reverse (5'-3')
m-Mlkl	AATTGTACTCTGGGAAATTGCCA	TCTCCAAGATTCCGTCCACAG
m-αSMA	GTCCCAGACATCAGGGAGTAA	TCGGATACTTCAGCGTCAGGA
m-Desmin	GTGGATGCAGCCACTCTAGC	TTAGCCGCGATGGTCTCATAC
m-Col1a1	GCTCCTCTTAGGGGCCACT	CCACGTCTCACCATTGGGG
m-Vimentin	CGTCCACACGCACCTACAG	GGGGGATGAGGAATAGAGGCT
m-Fsp1	TCCACAAATACTCAGGCAAAGAG	GCAGCTCCCTGGTCAGTAG
m-Pdgfrb	TTCCAGGAGTGATACCAGCTT	AGGGGGCGTGATGACTAGG
m-Timp1	GCAACTCGGACCTGGTCATAA	CGGCCCGTGATGAGAAACT
m-Loxl2	ATTAACCCCAACTATGAAGTGCC	CTGTCTCCTCACTGAAGGCTC
m-CCL1	GGCTGCCGTGTGGATACAG	AGGTGATTTTGAACCCACGTTT
m-CCL2	TTAAAAACCTGGATCGGAACCAA	GCATTAGCTTCAGATTTACGGGT
m-CCL3	TTCTCTGTACCATGACACTCTGC	CGTGGAATCTTCCGGCTGTAG
m-CCL4	TTCCTGCTGTTTCTCTTACACCT	CTGTCTGCCTCTTTTGGTCAG
m-CCL5	GCTGCTTTGCCTACCTCTCC	TCGAGTGACAAACACGACTGC
m-CX3CL1	ACGAAATGCGAAATCATGTGC	CTGTGTCGTCTCCAGGACAA
m-CCR1	CTCATGCAGCATAGGAGGCTT	ACATGGCATCACCAAAAATCCA
m-CCR2	ATCCACGGCATACTATCAACATC	CAAGGCTCACCATCATCGTAG
m-CXCR3	TACCTTGAGGTTAGTGAACGTCA	CGCTCTCGTTTTCCCCATAATC
m-CX3CR1	GAGTATGACGATTCTGCTGAGG	CAGACCGAACGTGAAGACGAG
m-TGFβ	CTCCCGTGGCTTCTAGTGC	GCCTTAGTTTGGACAGGATCTG
m-TNFα	CTGAACTTCGGGGTGATCGG	GGCTTGTCACTCGAATTTTGAGA
m-IL1β	GCAACTGTTCCTGAACTCAACT	ATCTTTTGGGGTCCGTCAACT
m-IL6	TAGTCCTTCCTACCCCAATTTCC	TTGGTCCTTAGCCACTCCTTC
m-iNOS	GTTCTCAGCCCAACAATACAAGA	GTGGACGGGTCGATGTCAC
m-IL12	ACTCTGCGCCAGAAACCTC	CACCCTGTTGATGGTCACGAC
m-Arg1	CTCCAAGCCAAAGTCCTTAGAG	AGGAGCTGTCATTAGGGACATC
m-Ym1	GGGCATACCTTTATCCTGAG	CCACTGAAGTCATCCATGTC
m-MMR	CTCTGTTCAGCTATTGGACGC	CGGAATTTCTGGGATTCAGCTTC
m-FIZZ1	GCCAGGTCCTGGAACCTTTC	GGAGCAGGGAGATGCAGATGAG
m-IL18	GACTCTTGCGTCAACTTCAAGG	CAGGCTGTCTTTTGTCAACGA
m-GAPDH	AGGTCGGTGTGAACGGATTTG	TGTAGACCATGTAGTTGAGGTCA
h-MLKL	AGGAGGCTAATGGGGAGATAGA	TGGCTTGCTGTTAGAAACCTG
h-αSMA	AAAAGACAGCTACGTGGGTGA	GCCATGTTCTATCGGGTACTTC
h-Desmin	TCGGCTCTAAGGGCTCCTC	CGTGGTCAGAAACTCCTGGTT
h-Vimentin	GACGCCATCAACACCGAGTT	CTTTGTCGTTGGTTAGCTGGT
h-Mmp2	TACAGGATCATTGGCTACACACC	GGTCACATCGCTCCAGACT
h-Col1a1	GTGCGATGACGTGATCTGTGA	CGGTGGTTTCTTGGTCGGT
h-Fsp1	GATGAGCAACTTGGACAGCAA	CTGGGCTGCTTATCTGGGAAG
h-GAPDH	CCACCTTTGACGCTGGG	CATACCAGGAAATGAGCTTGACA

Table S4. MLKL shRNA oligonucleotide templates utilized in lentivirus construction

Name	Forward (5'-3')	Reverse (5'-3')
Scramble	CCGGCCTAAGGTTAAGTCG<br>CCCTCGCTCGAGCGAGGGC<br>GACTTAACCTTAGGTTTTTG	GGCCGGATTCCAATTCAGCGGG<br>AGCGAGCTCGCTCCCGCTGAATT<br>GGAATCCAAAAAC
MLKL shRNA1	CCGGCCCAACATCCTGCGT<br>ATATTTCTCGAGAAATATAC<br>GCAGGATGTTGGGTTTTTG	AATTCAAAAACCCAACATCCTGC<br>GTATATTTCTCGAGAAATATACG<br>CAGGATGTTGGG
MLKL shRNA2	CCGGCCTCTGTGGATGAAA<br>TCTTAACTCGAGTTAAGATT<br>TCATCCACAGAGGTTTTTG	AATTCAAAAACCTCTGTGGATGA<br>AATCTTAACTCGAGTTAAGATTT<br>CATCCACAGAGG

Table S5. MLKL shRNA oligonucleotide templates utilized in AAV construction

Name	Forward (5'-3')	Reverse (5'-3')
Scramble	GGAAGTCGTGAGAAGTAGAA	GGAAGTCGTGAGAAGTAGAA
	TTAGTGAAGCCACAGATGTAA	TTACATCTGTGGCTTCACTAA
	TTCTACTTCTCACGACTTCC	TTCTACTTCTCACGACTTCC
MLKL	AGCAGAGAGATCCAGTTCAA	AGCAGAGAGATCCAGTTCAA
shRNA	CTAGTGAAGCCACAGATGTAG	CTACATCTGTGGCTTCACTAG
	TTGAACTGGATCTCTCTGCT	TTGAACTGGATCTCTCTGCT
